# Trends and inequalities in thinness and obesity among Chinese children and adolescents: evidence from seven national school surveys between 1985 and 2019

**DOI:** 10.1016/S2468-2667(24)00211-1

**Published:** 2024-10-29

**Authors:** Xinli Song, Bin Zhou, Sarah Baird, Chunling Lu, Majid Ezzati, Li Chen, Jieyu Liu, Yi Zhang, Ruolin Wang, Qi Ma, Jianuo Jiang, Yang Qin, Ziqi Dong, Wen Yuan, Tongjun Guo, Zhiying Song, Yunfei Liu, Jiajia Dang, Peijin Hu, Yanhui Dong, Yi Song, Jun Ma, Susan M Sawyer

**Affiliations:** Institute of Child and Adolescent Health & School of Public Health, Peking University, National Health Commission Key Laboratory of Reproductive Health, Beijing, China; School of Public Health, Imperial College London, London, UK; Department of Global Health, Milken Institute School of Public Health, George Washington University, Washington DC, USA; Division of Global Health Equity, Brigham and Women’s Hospital, Boston, MA, USA; School of Public Health, Imperial College London, London, UK; Institute of Child and Adolescent Health & School of Public Health, Peking University, National Health Commission Key Laboratory of Reproductive Health, Beijing, China; Centre for Adolescent Health, Royal Children’s Hospital, Parkville, VIC, Australia; Department of Paediatrics, Faculty of Medicine, Dentistry and Health Sciences, University of Melbourne, Parkville, VIC, Australia; Murdoch Children’s Research Institute, Parkville, VIC, Australia

## Abstract

**Background:**

There are little recent data in China regarding contemporary nutritional inequities among children and adolescents, particularly in relation to urban–rural residence and regional socioeconomic status (SES). We aim to assess inequalities in thinness and obesity in Chinese children and adolescents.

**Methods:**

Weight and height measurements for 1 677 261 children and adolescents aged 7–18 years were obtained from seven cycles of the Chinese National Surveys on Students Constitution and Health (1985, 1995, 2000, 2005, 2010, 2014, and 2019). Sex-specific BMI-for-age Z scores were applied to define thinness (Z scores <–2SD) and obesity (Z scores >+2SD). Urban–rural classification came from the Statistical Urban and Rural Division Code, and gross domestic product (GDP) per capita in the province in which the school was situated was used as a proxy for SES. T1 represented the provinces with the most disadvantaged SES and T3 represented the provinces with the most advantaged SES. General linear regression models assessed correlations between prevalence and GDP per capita, with projections to 2030 derived from best-fitting models.

**Findings:**

The mean prevalence of obesity rose from 0·10% (95% CI 0·09 to 0·11) in 1985 to 8·25% (8·13 to 8·37) in 2019, whereas thinness prevalence decreased from 8·49% (8·41 to 8·58) to 3·37% (3·29 to 3·45). High SES provinces exhibited a significant drop in obesity prevalence from 2014 (8·42% [8·19 to 8·65]) to 2019 (7·73% [7·52 to 7·95]). Nationally, the prevalence of obesity was consistently higher in urban areas than in rural areas for both sexes from 1985 to 2019; however, a greater prevalence of obesity was observed in rural than urban girls residing in T3 regions in 2019 (urban–rural gap: –0·37% [–0·07 to –0·80]). Rural boys had a higher prevalence of thinness than their urban counterparts across all survey waves, with the exceptions of 1985 and 1995. For girls, no significant urban–rural gap in thinness was observed in the most recent survey in 2019 (–0·10% [–0·24 to 0·04]). From 1985 to 2014, boys and girls from high SES regions had a higher risk of obesity and a lower risk of thinness than those from low SES regions. However, in 2019, a nationwide shift occurred, and the T3–T1 difference in obesity approached or went below zero for boys (–0·49% [–1·02 to 0·04]) and girls (–0·68% [–1·00 to –0·35]). T3–T1 differences in thinness also approached zero for boys (–0·46% [–0·77 to –0·14]) and girls (–0·14% [–0·43 to 0·15]). The projected estimates to 2030 for urban–rural obesity gaps (boys: –1·00% [–2·65 to 0·65]; girls: –2·88% [–6·91 to 1·15]) and T3–T1 obesity differences (boys: –8·88% [–13·76 to –4·01]; girls: –8·82% [–12·78 to –4·85]) were both negative, with forecasted estimates for urban–rural gaps and T3–T1 differences in thinness prevalence in 2030 close to zero for both boys and girls.

**Interpretation:**

China’s socioeconomic development continues to influence within-country inequities regarding the regional distribution of child and adolescent weight according to urban–rural location and regional SES. Contemporary Chinese children and adolescents in socioeconomically disadvantaged regions and rural areas constitute a vulnerable population facing nutritional risk, but from obesity rather than thinness. Disrupting projected inequities in obesity will require extensive preventive investments.

**Funding:**

National Natural Science Foundation of China, Beijing Natural Science Foundation, Peking University Talent Introduction Program Project, and Clinical Medicine Plus X-Young Scholars Project of Peking University.

## Introduction

The effects of obesity and thinness in childhood and adolescence have long-term health repercussions.^1^ Globally over the past three decades, a predominant pattern of undernutrition has shifted to one of overnutrition, linked to socioeconomic development.^1–4^ In China, which is home to more than 10% of the world’s population of children and adolescents,^5^ there has been a notable increase in the prevalence of obesity. Data from Chinese school students show obesity rising from 0·1% in 1985 to 6·4% in 2014,^6^ along with a decline in thinness from 8·5% to 4·2%.^7^

An obesity transition model has been proposed to describe discernible patterns of the spread of obesity within various demographic subpopulations within a country over time.^8^ Over the past few decades, like many low-income and middle-income countries (LMICs) in south and southeast Asia and sub-Saharan Africa, China has had higher obesity prevalence in urban and high socioeconomic status (SES) regions, whereas thinness is more prevalent in rural and low SES regions.^4,9,10^ Conversely, in a number of industrialised high-income countries (HICs), including Sweden,^11^ the USA,^12^ the UK,^13^ and Australia,^10^ school-aged children in rural and low SES regions commonly have a higher obesity prevalence, with a more uniform prevalence of thinness by region. Although distinct patterns between LMICs and HICs have been identified, little investigation has focused on the dynamic process involving the apparent shift between these two patterns as national wealth rises.

In China, there has been gradual narrowing of urban–rural disparities in height, BMI, obesity, and thinness.^7–9,14–16^ In 2021, the Chinese Government released an Implementation Plan for the Prevention and Control of Childhood Obesity, which set goals for nationwide and provincial-level prevention and control of the obesity epidemic by 2030.^17^ However, there have been no recent reports of the prevalence of child and adolescent obesity and thinness in China,^14^ and little research elsewhere has systematically tracked evolving patterns of regional nutrition disparities.

This study used 34 years of growth data from school-aged children and adolescents spanning seven waves (1985–2019) of the Chinese National Surveys on Students Constitution and Health (CNSSCH). This period has been characterised by remarkable growth in gross domestic product (GDP) that has seen China transition from a low-income to an upper-middle-income country. The objectives of this study were to systematically assess trends in obesity and thinness prevalence at both national and subnational levels, with a focus on urban–rural location and regional SES strata; and examine trends (including projections) in inequalities of obesity and thinness, in terms of urban–rural location and area-level SES, to understand how these disparities have evolved (and might continue to evolve), in response to growing national wealth.

## Methods

### Study design and participants

The CNSSCH is an extensive, nationwide school-based cross-sectional survey, conducted in 30 mainland provinces, using a multistage, stratified cluster sampling approach. This study focused on children and adolescents aged 7–18 years in China. Conducted at 5-year intervals since 1985, the most recent survey was in 2019. For each wave, each province was regarded as an independent subpopulation, and the provincial school health institutes gathered a representative sample of the entire province. Schools in each province were evenly selected based on the affluence level (upper, moderate, and low) of the prefecture-level city in which the school was located. A highly consistent sample was drawn within each province, ensuring a uniform approach across all regions. Participants were identified through stratified cluster sampling, with clusters randomly chosen from grades 1–12 in the selected primary and secondary schools. The sampling and investigation protocol have been consistent across all waves, as previously outlined.^7,9,18,19^ Eligible participants and their families needed to have lived locally for at least 1 year, and all measurements followed the same procedures with the same type of apparatus.^20^ Both children and parents provided written informed consent. The study protocols were approved by the Medical Research Ethics Committee of Peking University Health Science Center (IRB00001052–19095).

The present study combined data from seven consecutive CNSSCH cycles (1985, 1995, 2000, 2005, 2010, 2014, and 2019). Participants of Han ethnicity from 30 provinces, autonomous regions, and municipalities (referred to as provinces hereafter) in mainland China were included in the main analysis. Data were not collected from Hong Kong, Macau, or Taiwan, and the sample from Tibet was excluded due to its ethnic composition being predominantly of people from minority backgrounds. Participants with incomplete data on sex, age, setting, region, height, or bodyweight were excluded (<1%). The final sample consisted of 1 677 261 participants.

### Procedures

A detailed survey gathered individual demographic data. At all survey cycles, a complete medical examination, including standing height (cm) and weight (kg), was conducted by trained technicians as previously described.^7,9,18,19^ We derived sex-specific BMI-for-age Z scores using 2007 WHO growth references to define four mutually exclusive BMI categories: thinness (Z scores ≤median–2SD), normal weight (Z scores >median–2SD and ≤median+1SD), overweight (Z scores >median+1SD and ≤median+2SD), and obesity (Z scores >median+2SD).^21^

For each survey, we incorporated GDP per capita, at both national and provincial levels. This indicator was derived from statistical yearbooks released by the National and Provincial Bureau of Statistics of China.^22^ We obtained GDP per capita data (¥) and calculated GDP per capita (US$) as an indicator of economic perform-ance based on the purchasing power parity at the corresponding survey year.^23^ We then merged individual data from the CNSSCH with year-specific socioeconomic indicators at both provincial and national levels.

The foundational framework for urban and rural categorisation is found in the Statistical Urban and Rural Division Code, released by the National Bureau of Statistics.^24^ Within this framework, three primary classifications are delineated: cities, towns, and villages. This study used the education statistics’ urban–rural categorisation, where urban areas exclusively refer to cities, and rural areas includes towns and villages.^25^ The household registration system in China, commonly referred to as the *hukou* system, mandates that every Chinese citizen undergoes formal and continuous registration with the *hukou* police department from birth.^26^ This system means that each resident can ascertain their urban or rural resident status based on their registered residence in the *hukou* system. In accordance with the 2020 National Development of Education Report, there are 61·06 million (39·04%) primary and lower secondary school students enrolled in schools located in rural areas, and 95·33 million (60·96%) in urban areas.^27^ GDP per capita in the province where the school was situated was used as a proxy for SES. Based on quartiles of GDP per capita within the nationwide distribution in each survey year, the 30 provinces were divided into three groups: the first quartile, denoted as T1, represents provinces with the most disadvantaged SES; the second and third quartiles, denoted as T2, represent provinces with mid-level SES; the fourth quartile, denoted as T3, represents provinces with the most advantaged SES. This approach enabled us to obtain the SES of each province specific to each survey year.

### Statistical analysis

We present sample sizes and characteristics of participants from 1985 to 2019. Continuous variables are presented as means with 95% CIs, categorical variables are shown as numbers and percentages. The Mann–Kendall trend test examined trends over time. Difference of prevalences between two adjacent surveys was examined with χ^2^ tests. The prevalence of obesity and thinness at the subnational level was calculated separately for urban–rural residence and regional SES strata for both sexes. To assess annual changes, we computed the absolute difference in mean prevalence between two adjacent surveys and divided it by the number of years separating the two surveys. The urban–rural gap, indicating differences in prevalence rates between urban and rural areas, was a key indicator of inequalities in obesity and thinness among children and adolescents. The T3–T1 difference, reflecting disparities between the most socioeconomically advantaged and disadvantaged regions, was used to quantify SES-related inequalities.

For a detailed understanding of the urban–rural gradient in the 2019 survey, we used a heatmap to illustrate the urban–rural gap in obesity and thinness prevalence for each province. To examine the evolving patterns of within-country regional wealth gradients, scatter plots with fitted linear lines explored associations of provincial GDP per capita with obesity and thinness prevalence, specific to sex. Correlation coefficients *r* and corresponding p values were calculated using general linear regression models. We used multinomial (renormalised logistic) regression analyses to forecast the prevalence of each BMI category, using data points post-2000 to capture more recent trends.^28^ This approach ensures that the combined prevalence of all categories equals 100% annually and enables the estimation of non-linear trends in BMI category prevalence. Regressions were conducted for subgroups based on urban–rural divide and regional SES to derive projections for the urban–rural gap and the T3–T1 difference in obesity and thinness for both sexes in 2025 and 2030. To accommodate uncertainty, we used bootstrap resampling approaches on the datasets (1000 iterations) and reported the mean along with the 95% CIs (derived from the 2·5 and 97·5 percentiles of the bootstrapped values) for all estimates. In the China Economic and Social Development Mid- to Long-Term Goals, Strategies, and Paths Report released by State Information Center of China in 2021,^29^ the GDP per capita for China is forecasted to reach $12 050·08 in 2025 and $15 306·19 in 2030. We fitted national GDP per capita and urban–rural disparities, and T3–T1 difference in obesity and thinness for both sexes. Specific formulas and parameters are shown in the appendix 2 (p 19). In addition, the strategies and results of subgroup analysis and sensitivity analysis are presented in the appendix 2 (p 3). All statistical tests were two sided, and a significance level of p<0·05 was used to assess statistical significance. All analyses were conducted in R software version 4.2.2.

### Role of the funding source

The funder of the study had no role in study design, data collection, data analysis, data interpretation, or writing of the report.

## Results

Across the seven surveys, there was little change in the distribution of participants by sex, age, urban–rural residence, and regional SES strata (p>0·05; table). Over this period, GDP per capita climbed from $295 to $10 276. At the national level, the prevalence of obesity rose from 0·10% (95% CI 0·09–0·11) to 8·25% (8·13–8·37), whereas the prevalence of thinness decreased from 8·49% (8·41–8·58) to 3·37% (3·29–3·45), with a prominent trend towards obesity (p<0·01; table, appendix 2 p 4). Consistent findings were observed across all sex and urban–rural location subgroups at the provincial level (appendix 2 p 23).

**Figure 1:**
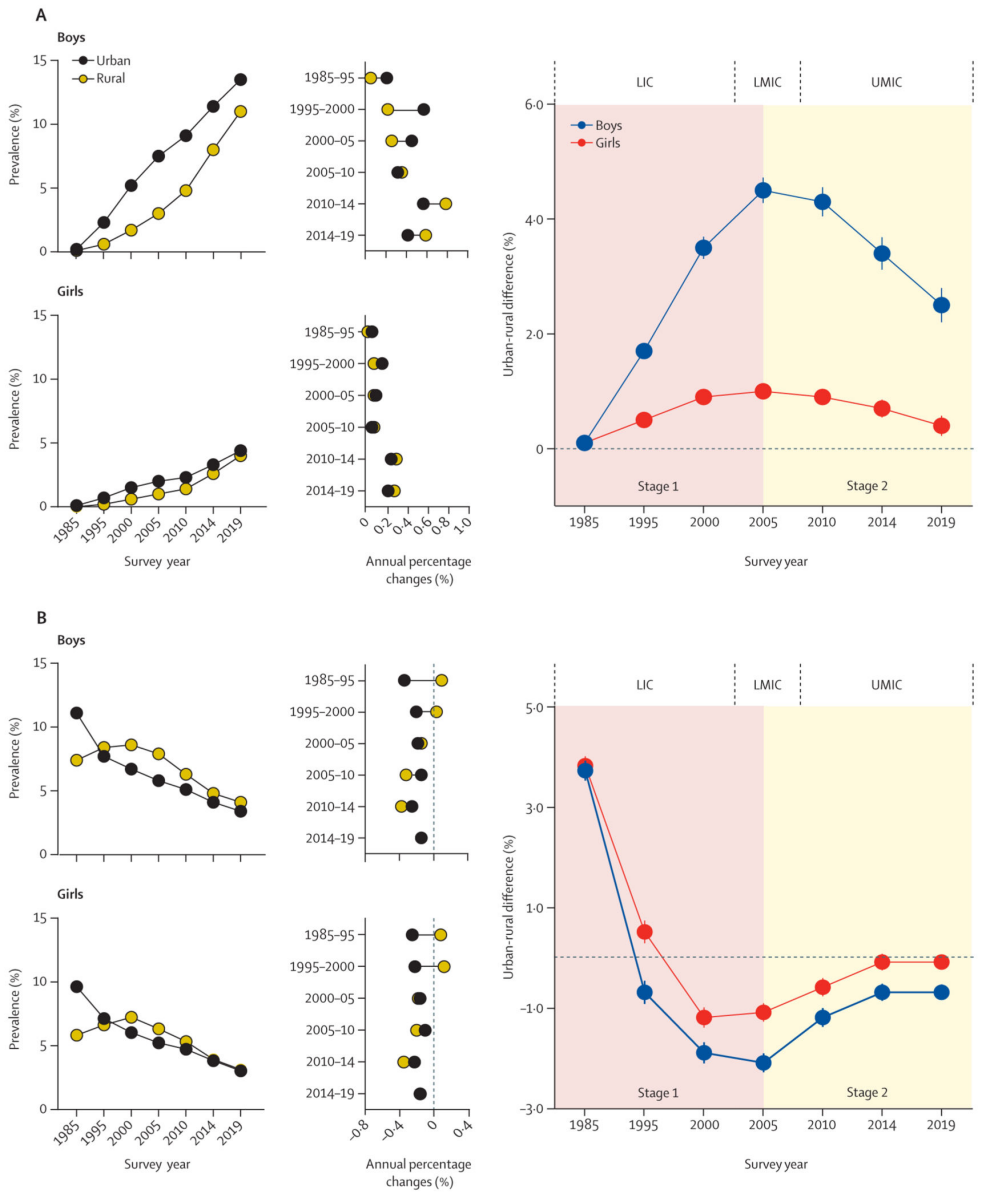
Temporal trends and inequalities in childhood and adolescent obesity (A) and thinness (B) by urban–rural residence, for boys and girls, 1985–2019 Stage 1 and stage 2 correspondingly signify the initial widening and subsequent narrowing of urban–rural disparities. LICs=low-income countries. LMICs=lower-middle-income countries. UMICs=upper-middle-income countries.

From 1985 to 2014, the mean prevalence of obesity among urban children and adolescents was consistently higher than those of rural residency (figure 1, appendix 2 pp 5–6). With the exception of 1985 and 1995, rural children persistently exhibited greater prevalence of thinness compared with their urban counterparts (figure 1, appendix 2 pp 5–6). Over time, there was a trend of initially widening followed by narrowing of the urban–rural gap in obesity and thinness, with peak levels of inequity observed in 2005 for both obesity (2·74% [95% CI 2·59 to 2·89]) and thinness (–1·64% [–1·84 to –1·44]; appendix 2 p 5). These findings were similar in boys and girls (figure 1, appendix 2 p 6).

**Figure 2:**
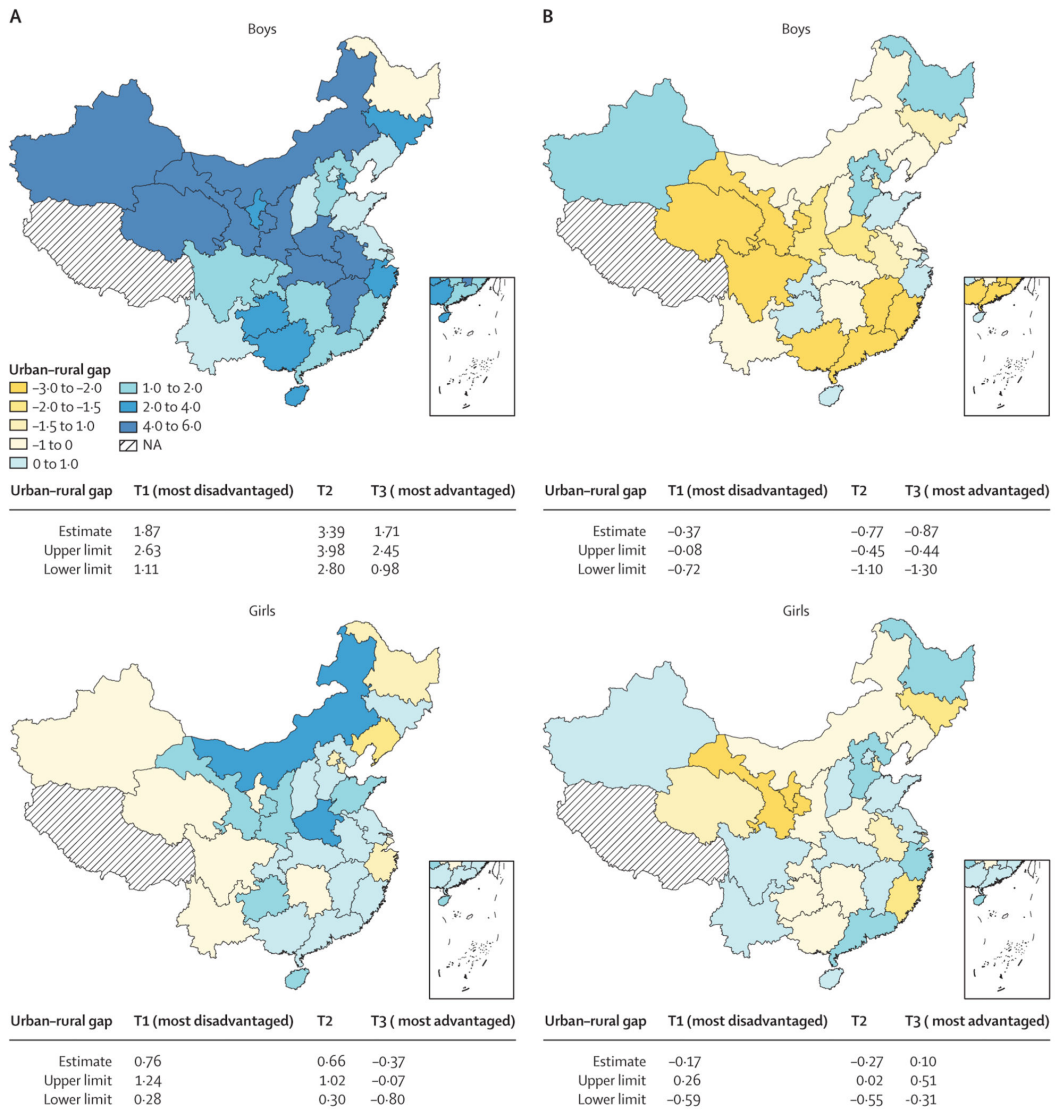
Urban–rural gap in obesity (A) and thinness (B) at the subnational level, for boys and girls in 2019 The estimates and 95% CIs of urban–rural gaps in obesity and thinness prevalence were presented, stratified by regional socioeconomic status. T1, the most disadvantaged socioeconomic status regions; T2, the moderate socioeconomic status regions; T3, the most advantaged socioeconomic status regions. NA=not applicable.

In 2019, unlike at the national level where obesity prevalence among girls living in urban areas exceeded that of girls in rural areas (0·40% [95% CI 0·16 to 0·64]), at the provincial level almost half of the provinces exhibited higher obesity prevalence among girls in rural areas compared with their urban counterparts, especially in provinces with high SES (girls’ urban–rural obesity gap in T3: –0·37% [–0·80 to –0·07]; figure 2, appendix 2 pp 4–6). Boys residing in urban areas were more likely to have obesity than those in rural area**s**, both nationally (urban–rural obesity gap: 2·52% [2·13 to 2·92]) and in most provinces (figure 2, appendix 2 pp 5–6). In 2019, there were statistically insignificant differences in thinness prevalence among girls residing in rural and urban areas, with consistent findings observed nationally and in most provinces (urban–rural thinness gap: –0·14% [–0·34 to 0·07]; figure 2, appendix 2 pp 5–6). By contrast, boys residing in rural areas were more likely to have thinness than their urban counterparts, both nationally (urban–rural thinness gap: –0·69% [–0·92 to –0·46]) and in most provinces.

**Figure 3:**
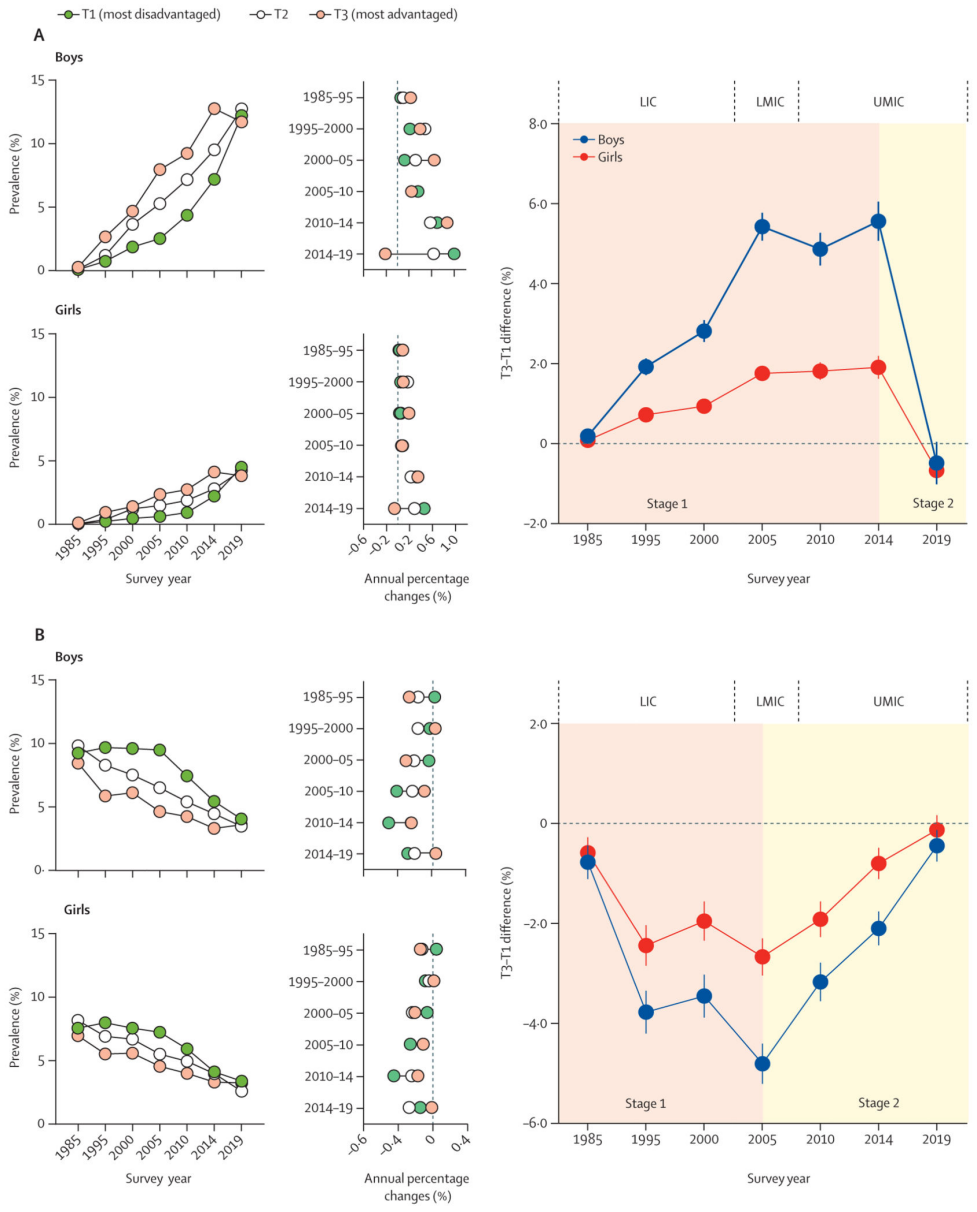
Temporal trends and inequalities in childhood and adolescent obesity (A) and thinness (B) by regional socioeconomic status, for boys and girls, 1985–2019 Stage 1 and stage 2 correspondingly signify the initial widening and subsequent narrowing of T3–T1 differences: T1, the most disadvantaged socioeconomic status regions; T2, the moderate socioeconomic status regions; T3, the most advantaged socioeconomic status regions. LICs=low-income countries. LMICs=lower-middle-income countries. UMICs=upper=middle-income countries.

From 1985 to 2019, there was an upward trend observed for obesity in the T1 and T2 regions; however, in the T3 regions, a significant decline was observed from 2014 (8·42% [95% CI 8·19–8·65]) to 2019 (7·73% [7·52–7·95]; figure 3, appendix 2 pp 6, 10). Both the T1 and T2 regions displayed a downward trend for thinness from 1985 to 2019 with a non-statistically significant rise observed in the T3 regions from 2014 (3·35% [3·20–3·50]) to 2019 (3·46% [3·32–3·61; figure 3, appendix 2 pp 6, 10). In addition, from 1985 to 2019, there has been an initial widening and subsequent narrowing in the T3–T1 difference with regards to obesity and thinness prevalence, for both sexes. Similar trends were found in the urban and rural subgroups (appendix 2 pp 17–18).

**Figure 4:**
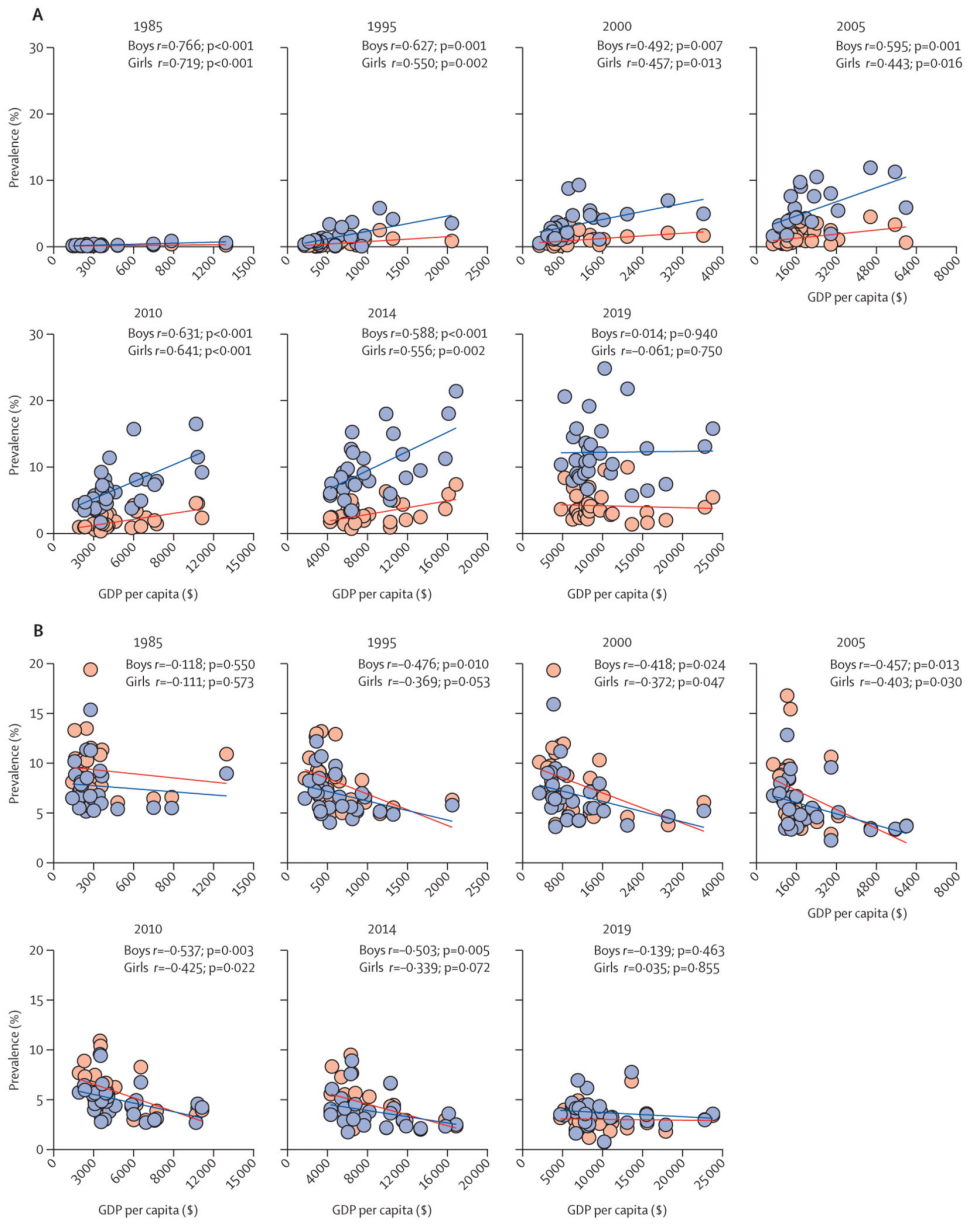
The regional wealth gradient in the prevalence of obesity (A) and thinness (B) within a given survey year, for boys and girls, 1985–2019 Each dot represents a province (boys in blue and girls in red). *R* is the coefficient of association between provincial GDP per capita and prevalence of obesity and thinness, and p value is for the correlation index. GDP=gross domestic product.

At the national level, the T3–T1 difference in obesity prevalence consistently remained above zero between 1985 and 2014; however, in 2019, the T3–T1 difference in obesity was equal to or below zero for both sexes (–0·60% [95% CI –0·91 to –0·29]), boys (–0·49% [–1·02 to 0·04]), and girls (–0·68 [–1·00 to –0·35]; figure 3, appendix 2 pp 6, 10). In addition, from 1985 to 2014, the T3–T1 difference in thinness prevalence consistently remained below zero. However, in 2019, there was a countrywide shift during which the T3–T1 difference in thinness was close to or equal to zero for both sexes (–0·30% [–0·52 to –0·08]), boys (–0·46% [–0·77 to –0·14]), and girls (–0·14% [–0·43 to 0·15]; figure 3, appendix 2 pp 6, 10). These observations at the provincial level support a substantial shift in the distribution of obesity and thinness among Chinese children and adolescents according to regional SES (figure 4). Between 1985 and 2014, there was a significant positive linear correlation (*r* values of 0·492–0·766 for boys and 0·443–0·719 for girls) between the prevalence of obesity and per capita GDP of each province, and a significant negative linear correlation (*r* values of –0·537 to –0·418 for boys and –0·425 to –0·339 for girls) between the prevalence of thinness and per capita GDP of each province. These correlations were accompanied by a yearly decrease in the correlation coefficient *r*, which showed no significant correlation in 2019 (figure 4).

**Figure 5:**
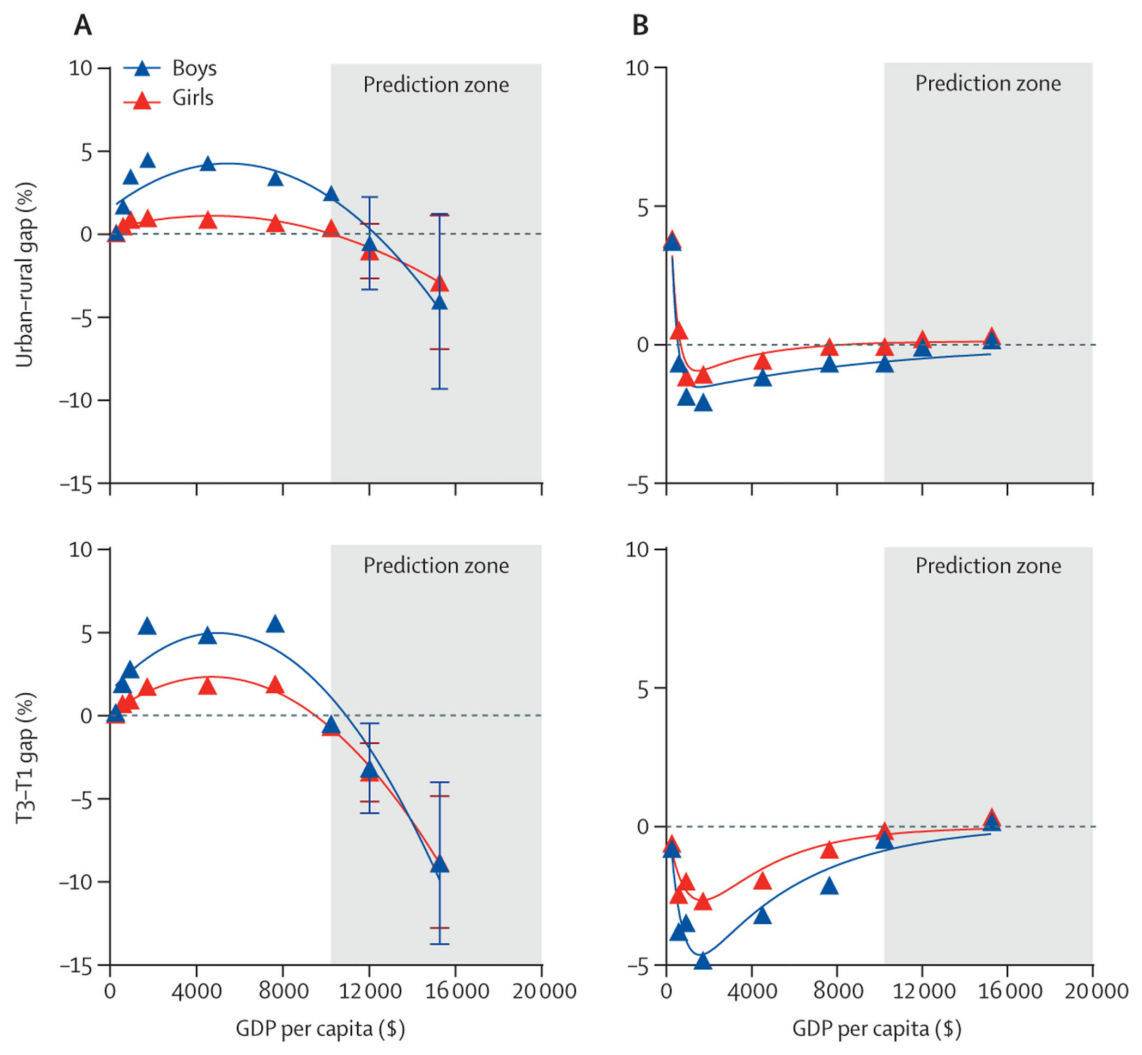
Projections of national GDP per capita with urban–rural gap and T3–T1 gap in obesity (A) and thinness (B), for boys and girls Each dot represents a survey year. *R*^2^ was calculated to evaluate the goodness-of-fit of the regression model. T1, the most disadvantaged socioeconomic status regions; T2, the moderate socioeconomic status regions; T3, the most advantaged socioeconomic status regions. GDP=gross domestic product.

Our projections indicate that the prevalence of childhood and adolescent obesity will continue to increase nationally in 2025 and 2030, irrespective of sex, in both urban and rural areas and across regions with varying SES (figure 5, appendix 2 pp 11–12). The projected estimates for urban–rural obesity gaps were negative for both 2025 (boys: –0·52% [95% CI –3·32 to 2·27]; girls: –1·00% [–2·65 to 0·65]), and 2030 (boys: –1·00% [–2·65 to 0·65]; girls: [–2·88% [–6·91 to 1·15]). The projected estimates for T3–T1 obesity differences were also negative for both 2025 (boys: –3·17% [–5·88 to –0·47]; girls: –3·41% [–5·17 to –1·66]) and 2030 (boys: –8·88% [–13·76 to –4·01]; girls: –8·82% [–12·78 to –4·85]**;** figure 5, appendix 2 pp 11–12). Additionally, the forecasted estimates for urban–rural gaps and T3–T1 differences in thinness prevalence for 2025 and 2030 are close to zero for both boys and girls (figure 5, appendix 2 pp 11–12).

## Discussion

The challenge of obesity inequity has garnered substantial attention in the context of the global increase in adult obesity between 1985 and 2017, which has been largely attributed to increases in rural areas.^3^ Most LMICs are witnessing rises in obesity at an equal or faster rate than in HICs,^3,30^ with concerns that the landscape of childhood and adolescent obesity is mirroring patterns observed in adults.^4,31^ Research indicates that within-country inequities in obesity in children and adolescents by urban–rural location and regional SES manifest distinct patterns in HICs (higher obesity prevalence in rural and low SES regions, thinness relatively uniform) and LMICs (higher obesity prevalence in urban and high SES regions, thinness more prevalent in rural and low SES regions). In this study, both patterns were evident nationally or regionally at different timepoints.

Between 1985 and 2014, our findings are consistent with studies from most LMICs, such as South Africa^32^ and India^33^ (and other studies from China^18,19^), where the most affluent regions and urban areas typically have an elevated risk of obesity. In China, disproportionate reductions in poverty in urban areas compared with rural areas have resulted in widening income inequalities by region. In urban areas and socioeconomically advantaged provinces, food choices for children and adolescents have been shaped by greater availability, price, and marketing,^34^ which have probably contributed to the more rapid rise and higher prevalence of obesity within these regions. A notable finding from the most recent CNSSCH survey was that a nutritional pattern more comparable to HICs has now emerged in China. In 2019, rural girls from high SES regions were more likely to have obesity than their urban counterparts, and those living in the low SES provinces were at higher risk of obesity than their peers in the high SES provinces. Our earlier study using data up to the 2014 CNSSCH survey hinted at a possible shift; however, at that time, students in rural areas of only three affluent provinces had a higher risk of obesity.^16^ Our current findings are consistent with those in a smaller national cross-sectional survey that covered 11 provinces between 2017 and 2019, which found that the highest prevalence of obesity was in the western regions of China that are characterised by economic deprivation.^35^

Our projections suggest that these trends will only continue in the future without intensive preventive interventions. Contemporary rural students face comparable or heightened risk factors for excess bodyweight compared with their urban counterparts, especially around food choices,^36^ physical activity, and sedentary behaviours.^37^ A further challenge for rural students is more limited access to nutrition, health education, and health-care services. The swift catch-up and subsequent surpassing of childhood obesity rates in low SES regions compared with high SES regions in China since 2005 is likely to reflect overall improvements in living standards. Over this same period, there has been considerable funding of school health by provincial governments in socioeconomically advantaged regions. In provinces and cities such as Beijing, Guangdong, Zhejiang, and Jiangsu, investment in Health-Promoting Schools programmes has resulted in widespread commitment to nutrition and physical activity since 1995, consistent with WHO recommendations.^38,39^ In a 2018 national study, coverage of school health monitoring systems in Beijing, Guangdong, and Jiangsu surpassed 80%, emphasising that high SES regions can bring government attention and funding for school health.^40^ In our study, these same high SES provinces displayed a small declining trend in obesity in 2019. Although the extent to which these improvements can be directly attributed to the interventions remains unknown, our finding reinforces how dynamic these trends can be.

Thinness in China has been relatively overlooked in recent research on nutritional trends and inequalities, perhaps reflecting declining trends and recent low prevalence. It is noteworthy that between 1985 and 2000, an unexpected rise in thinness was observed in rural areas that had recently transitioned from extreme poverty. Some improvements in growth were seen, namely increased height, but there was no corresponding increase in weight, with lower BMI values, elevated thinness rates, and enhanced urban–rural disparities. Between 2000 and 2014, children and adolescents from low SES regions and rural areas in China faced a heightened risk of thinness compared with those in high SES regions and urban areas. This risk is even more pronounced in some LMICs where undernutrition remains the major burden of malnutrition, such as India^38^ and countries in sub-Saharan Africa.^39^ Indeed, the Chinese central Government has long invested in nutritional strategies to address undernutrition in rural areas. In addition to rising household income and declining poverty nationwide, specific programmes such as the China School Milk Program (2000) and a programme to improve student nutrition among rural students (issued by The State Council in 2011 and revised in 2022)^41^ might also have contributed to eliminating urban–rural inequalities in undernutrition.^42^ Notwithstanding these gains, substantial regional variations persisted in 2019, with thinness prevalence ranging from 0·72% to 7·34% by province, independent of provincial socioeconomic development. The coexistence of undernutrition and overnutrition in particular provinces in China means that these local governments must invest in double-duty actions that ensure the efforts to reduce thinness do not inadvertently contribute to overweight and obesity.^42^

This study has several strengths. First, its novelty in scale and scope facilitates systematic tracking of within-country inequalities in obesity and thinness over more than three decades, during which there have been dramatic changes in socioeconomic development in China. Using consistent sampling methods, the CNSSCH has recruited a large, representative sample, and obtained highly accurate measurements of height and weight that enhance the accuracy of our prevalence estimates. Second, our categorisation of urban and rural residence was determined using the Statistical Urban and Rural Division Code provided by the National Bureau of Statistics, which enhances the integration of our findings into policy. Finally, GDP per capita, the most commonly used socioeconomic indicator, was used to delineate the regional socioeconomic gradient, which facilitates the generalisability of our findings to other countries. Additional sensitivity analyses to examine the robustness of our findings were conducted using the value added of the primary sector as a percentage of GDP to define regional SES (in LMICs, the primary sector generally makes up a larger portion of the economy than in HICs), which yielded almost entirely consistent observations (appendix 2 p 16). Using multilevel logistic regression analysis, we observed nearly identical trends in the adjusted inequalities related to urban–rural location and regional SES, with closely aligned point estimates (appendix 2 pp 21–22, 24–25).

Some limitations also need to be considered. First, the CNSSCH surveys are school-based and inherently exclude children not attending school. High enrolment of contemporary Chinese school-age children since 2005 (≥95% of students receive at least 9 years of schooling) implies good representativeness of these surveys (appendix 2 p 20). Reduced enrolment in earlier waves might have underestimated nutritional disparities, as higher socioeconomic status was associated with higher likelihood of enrolment at that time. Second, it is important to exercise caution when extrapolating our findings to other ethnic groups, as this study only focused on those of Han ethnicity. Using data from China’s other ethnic groups from 2005 to 2019 (appendix 2 pp 13–15), we replicated the main analysis, which revealed similar trends in the regional distribution of obesity and thinness to those of the Han ethnic group in the main analysis. Third, due to sustained internal migration in China, there are differences between *hukou* (birth registration defined as urban or rural) and school enrolment records, which might obscure the regional characteristics our findings set out to explore. However, separate analyses (results not shown) showed that the proportion of rural or urban students in each school type exceeded 90% in more than 80% of schools, so we do not believe that this limitation is a major threat to our findings. This finding therefore suggests that China’s vision of equalising access to basic public education across different regions remains hindered by unequal educational investments and administrative barriers to social supports and educational resources imposed by the *hukou* system.^43^ Fourth, this study uses proxy measures of urban–rural residence and socioeconomic status, which might not fully capture their complexity and might thus limit the accuracy of our findings. China’s distinctive political landscape is marked by a dual urban–rural divide and decentralised provincial authority. One benefit of our approach is that pinpointing current and future high-risk areas for childhood and adolescent obesity and thinness enables more optimal resource allocation and targeting of effective interventions. Fifth, due to limitations in the number of time-series data points, we did not conduct an assessment of the accuracy of the predictive model. However, we used a large number of individual-level data points and produced uncertainty intervals for the expected estimates using bootstrapping. The impact of the COVID-19 pandemic might have led to underestimated obesity rates, and the anticipated reversal in obesity trends could be more substantial and imminent than our models predict. In addition, although the statistical methods used are suitable for addressing the research questions, the interpretation of results should consider potential limitations and assumptions inherent to each method, such as the assumption of linearity in regression models and the potential for type I error in hypothesis testing. Finally, it is important to acknowledge that our study’s reliance on the WHO 2007 growth standards might not fully account for the specific weight patterns of Chinese populations.

In conclusion, this study provides compelling new insights into the shifting landscape of obesity and thinness among Chinese children and adolescents, at both national and subnational levels. Although the effects of the COVID-19 pandemic on future wealth and weight remain uncertain, our projections signal an imminent reversal in the patterns of urban–rural and socioeconomic disparities, whereby future obesity burdens and the associated health challenges will be concentrated in rural areas and the most deprived regions. Despite reducing thinness in children and adolescents, considerable variation persists across provinces, with some regions having to respond to the dual challenge of both obesity and thinness. Beyond binary considerations of thinness or obesity, or of urban or rural regions, these findings highlight the need for policies to become nuanced in how they equitably enhance nutritional outcomes in children and adolescents, emphasising the urgency of crafting tailored nutrition policies and strategies for diverse socioeconomic environments and specific subpopulations.

## Supplementary Material

Appendix

## Figures and Tables

**Table:** Characteristics of participants from seven successive national surveys of CNSSCH

	1985 (n=409 946)	1995 (n=204 932)	2000 (n=209 167)	2005 (n=225 213)	2010 (n=208 136)	2014 (n=207 154)	2019 (n=212 713)	p for trend*
**Sex**								
Boys	205 100 (50·0%)	103 102 (50·3%)	104 555 (50·0%)	112 990 (50·2%)	104 093 (50·0%)	103 616 (50·0%)	106 703 (50·2%)	··
Girls	204 846 (50·0%)	101 830 (49·7%)	104 612 (50·0%)	112 223 (49·8%)	104 043 (50·0%)	103 538 (50·0%)	106 010 (49·8%)	0·999
**Age, years**								
7–9	102 678 (25·0%)	49 148 (24·0%)	52 355 (25·0%)	55 745 (24·8%)	52 038 (25·0%)	51 902 (25·1%)	53 916 (25·3%)	0·085
10–12	102 715 (25·1%)	52 259 (25·5%)	52 481 (25·1%)	56 166 (24·9%)	52 108 (25·0%)	51 971 (25·1%)	53 937 (25·4%)	0·999
13–15	102 695 (25·1%)	51 953 (25·4%)	52 038 (24·9%)	56 163 (24·9%)	52 067 (25·0%)	52 061 (25·1%)	53 310 (25·1%)	0·999
16–18	101 858 (24·8%)	51 572 (25·2%)	52 293 (25·0%)	57 139 (25·4%)	51 923 (24·9%)	51 220 (24·7%)	51 550 (24·2%)	0·230
**Residence**								
Urban	204 727 (49·9%)	103 741 (50·6%)	105 094 (50·2%)	113 439 (50·4%)	103 982 (50·0%)	103 639 (50·0%)	106 706 (50·2%)	··
Rural	205 219 (50·1%)	101 191 (49·4%)	104 073 (49·8%)	111 774 (49·6%)	104 154 (50·0%)	103 515 (50·0%)	106 007 (49·8%)	0·999
**Regional ** **SES**								
T1 (most disadvantaged)	102 762 (25·1%)	60 590 (29·6%)	57 043 (27·3%)	61 753 (27·4%)	57 247 (27·5%)	57 287 (27·7%)	56 445 (26·5%)	0·764
T2	190 114 (46·4%)	86 027 (42·0%)	83 670 (40·0%)	101 686 (45·2%)	93 342 (44·8%)	92 960 (44·9%)	97 284 (45·7%)	0·764
T3 (most advantaged)	117 070 (28·6%)	58 315 (28·5%)	68 454 (32·7%)	617 74 (27·4%)	57 547 (27·6%)	56 907 (27·5%)	58 984 (27·7%)	0·368
**Nutritional status**								
Thinness	34 811 (8·5%)	15 258 (7·4%)	14 891 (7·1%)	14 173 (6·3%)	11 098 (5·3%)	8618 (4·2%)	7169 (3·4%)	0·003
Obesity	405 (0·1%)	2004 (1·0%)	4761 (2·3%)	7572 (3·4%)	9151 (4·4%)	13 186 (6·4%)	17 548 (8·2%)	0·003
**Socioeconomic indicator** †								
GDP per capita ($)	295 (205–328)	610 (392–763)	959 (605–1294)	1754 (1223–2283)	4551 (3501–6157)	7679 (5787–10291)	10 276 (6988–11 162)	0·003

Data shown are n (%) or median (IQR). CNSSCH=Chinese National Surveys on Students Constitution and Health. GDP=gross domestic product. SES=socioeconomic status.

* p for trend was examined by Mann–Kendall trend test.

† Socioeconomic indicators were presented as the national-level values and the IQR of province-level values within the nationwide distribution for each survey year.

## Data Availability

All data in this article can be shared. Requests with appropriate ethics board approvals and study protocols will be assessed by the Institute of Child and Adolescent Health, Peking University.

## Research in context

### Evidence before this study

We searched PubMed on May 20, 2024 using the broad terms (“urban–rural” OR “regional socioeconomic status” OR “regional wealth gradient” OR “public health policies”) AND (“nutritional status” OR “nutritional problems” OR “nutritional burdens” OR “nutritional transition” OR (“obesity” OR “thinness” OR “overweight” OR “undernutrition”)) AND “trends” AND (“Chinese” OR “China”) AND (“youth” OR “children” OR “adolescents” OR “student”) for articles published in English. Some studies on within-country inequity patterns of regional distribution of obesity and thinness among Chinese children and adolescents have focused on urban–rural location and regional socioeconomic status (SES), showing that those in socioeconomically advantaged provinces and urban areas have a greater risk of obesity and a lower risk of thinness compared with those in socioeconomically disadvantaged provinces and rural areas, with these differences gradually shrinking over time. However, these studies are limited in scope as they often only examine a single timepoint, only address urban–rural disparities rather than SES, and rarely extend beyond 2014. More recent trends are unknown.

### Added value of this study

Contemporary Chinese children and adolescents continue to exhibit a pattern of nutritional inequality that is similar to that of most low-income and middle-income countries (LMICs), albeit less pronounced than previously. In 2019, the urban–rural disparity and the gap in obesity between high and low SES regions had further narrowed, even reversing at the national and subnational level; disparities in thinness across regions also decreased, or even disappeared entirely. Our projections indicate that future patterns of childhood and adolescent obesity in China are poised to be concentrated in rural and low SES regions, whereas thinness will be evenly distributed, resembling patterns observed in many industrialised high-income countries (HICs). Identifying high-risk areas of childhood obesity and thinness within a country is beneficial for regional policy makers in formulating obesity-related objectives and equitably allocating resources for preventive actions and health services.

### Implications of all the available evidence

Over the past three decades, rapid socioeconomic development in China has been accompanied by a shift in the pattern of nutritional inequalities related to urban–rural residence and regional SES from that typical of most LMICs to one more akin to that of industrialised HICs. Effectively implementing interventions targeting young people living in socioeconomically disadvantaged regions and rural areas is required to curb the continued escalation of obesity and its related health burdens of non-communicable diseases. The coexistence of both undernutrition and overnutrition in specific provinces also highlights the importance of respective local governments taking double-duty actions in these regions.
