# Supplementary material for: Trends and inequalities in thinness and obesity among Chinese children and adolescents: evidence from seven national school surveys between 1985 and 2019
Source: Lancet Public Health. Author manuscript; Available in PMC 2024 Nov 8. (PMC7616785; doi:10.1016/S2468-2667(24)00211-1)
Supplement: Appendix [file EMS199744-supplement-Appendix.pdf]

THE LANCET
Public Health

## Supplementary appendix 2

This appendix formed part of the original submission and has been peer reviewed. We post it as supplied by the authors.

Supplement to: Song X, Zhou B, Baird S, et al. Trends and inequalities in thinness and obesity among Chinese children and adolescents: evidence from seven national school surveys between 1985 and 2019. *Lancet Public Health* 2024; published online Oct 28. https://doi.org/10.1016/S2468-2667(24)00211-1.

# Appendix

**Trends and inequalities in thinness and obesity among Chinese children and adolescents: evidence from seven national school surveys between 1985 and 2019**

**Authors:** Xinli Song[1], PhD; Bin Zhou[2], PhD; Sarah Baird[3], PhD; Chunling Lu[4], PhD; Majid Ezzati[2], PhD; Li Chen[1], PhD; Jieyu Liu[1], PhD; Yi Zhang[1], MS; Ruolin Wang[1], MS; Qi Ma[1], MS; Jianuo Jiang[1], MS; Yang Qin[1], MS; Ziqi Dong[1], MS; Wen Yuan[1], PhD; Tongjun Guo[1], PhD; Zhiying Song[1], PhD; Yunfei Liu[1], PhD; Jiajia Dang[1], PhD; Peijin Hu[1], PhD; Yanhui Dong[1*], PhD; Yi Song[1*], PhD; Jun Ma[1], PhD; Susan M Sawyer[5,6,7], MD

**Affiliations**:

[1] Institute of Child and Adolescent Health & School of Public Health, Peking University; National Health Commission Key Laboratory of Reproductive Health, Beijing, China

[2] School of Public Health, Imperial College London, London, UK

[3] Department of Global Health, Milken Institute School of Public Health, George Washington University, Washington DC, USA

[4] Division of Global Health Equity, Brigham and Women's Hospital, Boston, Massachusetts, USA

[5] Centre for Adolescent Health, Royal Children's Hospital, Parkville, Victoria, Australia

[6] The University of Melbourne, Faculty of Medicine, Dentistry and Health Sciences, Department of Paediatrics, Parkville, Victoria, Australia

[7] Murdoch Children’s Research Institute, Parkville, Victoria, Australia

***Correspondence**:

Yanhui Dong and Yi Song, Institute of Child and Adolescent Health & School of Public Health, Peking University, No. 38 Xueyuan Rd, Haidian District, Beijing 100191, China, Tel: +86-10-82801524, Fax: +86-10-82801178, Email: dongyanhui@bjmu.edu.cn or songyi@bjmu.edu.cn.

# Contents

Subgroup analyses and sensitivity analyses .......... 3
Table S1. The prevalence of obesity and thinness among Chinese children and adolescents for both sexes, 1985-2019 .......... 4
Table S2 The prevalence of obesity and thinness and the annual change, by urban-rural residence, 1985-2019 .......... 5
Table S3. The prevalence of obesity and thinness, by urban-rural residence and regional socioeconomic status for boys and girls, 1985-2019 .......... 6
Table S4. Urban-rural difference in prevalence of obesity and thinness from 1985 to 2019, by regional socioeconomic status for boys and girls .......... 8
Table S5. The prevalence of obesity and thinness, and annual change, by regional socioeconomic status strata, 1985-2019 10
Table S6. Projected prevalence of obesity and thinness in 2025 and 2030, by urban-rural location and sex .......... 11
Table S7. Projected prevalence of obesity and thinness in 2025 and 2030, by regional SES and sex .......... 12
Table S8. Characteristics of ethnic minority participants from four national surveys of CNSSCH from 2005 to 2019 .......... 13
Table S9. The prevalence of obesity and thinness among ethnic minority participants aged 7-18, by regional socioeconomic status, 2005-2019 .......... 14
Table S10. The prevalence of obesity and thinness among ethnic minority participants aged 7-18, by urban-rural location, 2005-2019 .......... 15
Table S11. The prevalence of obesity and thinness among participants of Han ethnic, by regional socioeconomic status, 1985-2019 .......... 16
Table S12. The prevalence of obesity and thinness among urban participants of Han ethnic, by regional socioeconomic status, 1985-2019 .......... 17
Table S13. The prevalence of obesity and thinness among rural participants of Han ethnic, by regional socioeconomic status, 1985-2019 .......... 18
Table S14. The formula and parameters of the fit model .......... 19
Table S15. Nine-year compulsory education enrolment rate for school-age children in China, 1990-2020 .......... 20
Table S16. Adjusted prevalence (%) of childhood and adolescent obesity and thinness for boys, by urban-rural location and regional SES .......... 21
Table S17. Adjusted prevalence (%) of childhood and adolescent obesity and thinness for girls, by urban-rural location and regional SES .......... 22
Figure S1. Secular trends in the prevalence of obesity and thinness at the provincial level, for both boys (a) and girls (b), stratified by urban-rural residence, 1985-2019 .......... 23
Figure S2. Absolute urban-rural and regional socioeconomic inequalities (%) in childhood adolescent obesity, by sex .......... 24
Figure S3. Absolute urban-rural and regional socioeconomic inequalities (%) in childhood adolescent thinness, by sex .......... 25

**Subgroup analyses and sensitivity analyses**

Three sensitivity analyses were conducted. We repeatedly calculated the T3-T1 difference in the prevalence of obesity and thinness, using the value added of primary sector as per cent of GDP (%) as a proxy indicator for regional SES. Generally, in less developed economies, the proportion of the economy accounted for by the primary sector is higher, which tends to indicate a lower regional SES. Given China's multi-ethnic nature, the main analyses only involved the Han ethnic group. To test the robustness of our findings, we included minority populations in the sensitivity analyses. Since the minority groups were only included in the CNSSCH cycles after 2005, and their distribution across provinces and between urban and rural areas is highly uneven, coupled with significant heterogeneity in their nutritional status compared to the Han population, we thus analyzed the data of minority groups separately rather than merging them with the Han dataset. Furthermore, we applied the adjusted the adjusted urban-rural and T3-T1 inequalities (and their 95% confidence intervals [CI]) to again test the robustness of our findings. Multilevel logistic regression analysis with random intercepts by province was applied, controlling for clustering effects at the province level. Using regression analysis, we were able to obtain (1) the adjusted likelihood (and their 95% CI) of childhood and adolescent obesity and thinness for each group by urban-rural location and regional SES; and (2) the adjusted urban-rural and T3-T1 inequalities (and their 95% CI).

In the subgroup analyses, estimates of T3-T1 differences in obesity and thinness prevalence, were separately conducted for urban and rural subgroups using GDP per capita as proxy indicators for regional SES, respectively.

**Table S1. The prevalence of obesity and thinness among Chinese children and adolescents for both sexes, 1985-2019**

| | Both sexes | | | | | | |
|---|---|---|---|---|---|---|---|
| | **1985** | **1995** | **2000** | **2005** | **2010** | **2014** | **2019** |
| Obesity | 0·10 (0·09, 0·11) | 0·98 (0·94, 1·02) | 2·28 (2·21, 2·34) | 3·36 (3·29, 3·44) | 4·40 (4·31, 4·48) | 6·37 (6·26, 6·47) | 8·25 (8·13, 8·37) |
| Thinness | 8·49 (8·41, 8·58) | 7·45 (7·33, 7·56) | 7·12 (7·01, 7·23) | 6·29 (6·19, 6·39) | 5·33 (5·24, 5·43) | 4·16 (4·07, 4·25) | 3·37 (3·29, 3·45) |

**Table S2 The prevalence of obesity and thinness and the annual change, by urban-rural residence, 1985-2019**

| | 1985 | 1995 | 2000 | 2005 | 2010 | 2014 | 2019 |
|---|---|---|---|---|---|---|---|
| **Obesity prevalence (95% CIs)** | | | | | | | |
| Urban | 0·16 (0·14, 0·18) | 1·53 (1·46, 1·61) * | 3·36 (3·25, 3·47) * | 4·72 (4·60, 4·84) * | 5·67 (5·53, 5·81) * | 7·39 (7·23, 7·55) * | 9·00 (8·83, 9·17) * |
| Rural | 0·04 (0·03, 0·05) | 0·41 (0·37, 0·45) * | 1·18 (1·11, 1·24) * | 1·98 (1·90, 2·06) * | 3·13 (3·02, 3·23) * | 5·34 (5·21, 5·48) * | 7·49 (7·33, 7·65) * |
| Urban-rural difference | 0·12 (0·10, 0·14) | 1·12 (1·04, 1·20) | 2·19 (2·06, 2·31) | 2·74 (2·59, 2·89) | 2·55 (2·37, 2·72) | 2·04 (1·83, 2·25) | 1·51 (1·27, 1·74) |
| **Thinness prevalence (95% CIs)** | | | | | | | |
| Urban | 10·38 (10·24, 10·51) | 7·39 (7·23, 7·55) * | 6·35 (6·20, 6·50) * | 5·48 (5·35, 5·61) * | 4·88 (4·75, 5·01) * | 3·94 (3·82, 4·06) * | 3·16 (3·06, 3·27) * |
| Rural | 6·61 (6·5, 6·72) | 7·50 (7·34, 7·66) * | 7·89 (7·73, 8·06) * | 7·12 (6·97, 7·27) * | 5·79 (5·64, 5·93) * | 4·38 (4·26, 4·51) * | 3·58 (3·47, 3·69) * |
| Urban-rural difference | 3·76 (3·59, 3·93) | -0·11 (-0·34, 0·11) | -1·54 (-1·76, -1·32) | -1·64 (-1·84, -1·44) | -0·91 (-1·10, -0·72) | -0·45 (-0·62, -0·27) | -0·41 (-0·57, -0·26) |
| | | **1985-1995** | **1995-2000** | **2000-2005** | **2005-2010** | **2010-2014** | **2014-2019** |
| **Annual percentage change in obesity prevalence (95% CIs)** | | | | | | | |
| Urban | - | 0·14 (0·13, 0·14) | 0·37 (0·34, 0·39) | 0·27 (0·24, 0·30) | 0·19 (0·15, 0·23) | 0·43 (0·38, 0·48) | 0·32 (0·28, 0·37) |
| Rural | - | 0·04 (0·03, 0·04) | 0·15 (0·14, 0·17) | 0·16 (0·14, 0·18) | 0·23 (0·20, 0·26) | 0·55 (0·51, 0·60) | 0·43 (0·39, 0·47) |
| **Annual percentage change in thinness prevalence (95% CIs)** | | | | | | | |
| Urban | - | -0·30 (-0·32, -0·28) | -0·21 (-0·25, -0·16) | -0·17 (-0·21, -0·14) | -0·12 (-0·16, -0·08) | -0·23 (-0·28, -0·19) | -0·15 (-0·19, -0·12) |
| Rural | - | 0·09 (0·07, 0·11) | 0·08 (0·03, 0·12) | -0·15 (-0·20, -0·11) | -0·27 (-0·31, -0·23) | -0·35 (-0·40, -0·30) | -0·16 (-0·19, -0·13) |

*Note*: Difference of prevalences between two adjacent surveys was examined by $\chi^2$ test, $^*P < 0.05$.

**Table S3. The prevalence of obesity and thinness, by urban-rural residence and regional socioeconomic status for boys and girls, 1985-2019**

| | Boys | | | | | | | Girls | | | | | | |
|---|---|---|---|---|---|---|---|---|---|---|---|---|---|---|
| | **1985** | **1995** | **2000** | **2005** | **2010** | **2014** | **2019** | **1985** | **1995** | **2000** | **2005** | **2010** | **2014** | **2019** |
| **Obesity** | | | | | | | | | | | | | | |
| **Urban-rural location** | | | | | | | | | | | | | | |
| Urban | 0·24 (0·21, 0·27) | 2·34 (2·21, 2·47) | 5·24 (5·05, 5·43) | 7·46 (7·24, 7·67) | 9·06 (8·82, 9·31) | 11·43 (11·15, 11·70) | 13·54 (13·25, 13·83) | 0·08 (0·06, 0·10) | 0·73 (0·65, 0·80) | 1·49 (1·38, 1·59) | 1·97 (1·86, 2·09) | 2·27 (2·15, 2·40) | 3·35 (3·19, 3·50) | 4·39 (4·21, 4·56) |
| Rural | 0·05 (0·04, 0·06) | 0·60 (0·53, 0·67) | 1·73 (1·62, 1·84) | 3·00 (2·86, 3·15) | 4·84 (4·66, 5·03) | 8·05 (7·82, 8·28) | 11·02 (10·75, 11·28) | 0·03 (0·02, 0·04) | 0·22 (0·18, 0·26) | 0·63 (0·56, 0·7) | 0·95 (0·87, 1·03) | 1·41 (1·31, 1·51) | 2·63 (2·49, 2·77) | 3·98 (3·82, 4·15) |
| Urban-rural difference | 0·19 (0·16, 0·22) | 1·74 (1·59, 1·88) | 3·51 (3·29, 3·73) | 4·45 (4·19, 4·71) | 4·22 (3·91, 4·53) | 3·38 (3·02, 3·74) | 2·52 (2·13, 2·92) | 0·05 (0·03, 0·07) | 0·51 (0·43, 0·59) | 0·86 (0·74, 0·98) | 1·02 (0·88, 1·16) | 0·86 (0·70, 1·03) | 0·72 (0·51, 0·93) | 0·40 (0·16, 0·64) |
| **Regional socioeconomic status** | | | | | | | | | | | | | | |
| T1 (most disadvantaged) | 0·09 (0·06, 0·11) | 0·74 (0·64, 0·83) | 1·86 (1·70, 2·02) | 2·52 (2·34, 2·69) | 4·35 (4·12, 4·59) | 7·17 (6·87, 7·46) | 12·18 (11·80, 12·56) | 0·02 (0·01, 0·03) | 0·21 (0·16, 0·26) | 0·45 (0·38, 0·53) | 0·59 (0·51, 0·68) | 0·91 (0·80, 1·02) | 2·21 (2·04, 2·38) | 4·49 (4·25, 4·73) |
| T2 | 0·10 (0·08, 0·12) | 1·19 (1·09, 1·30) | 3·64 (3·46, 3·82) | 5·26 (5·07, 5·46) | 7·15 (6·92, 7·39) | 9·49 (9·23, 9·76) | 12·71 (12·41, 13·00) | 0·04 (0·03, 0·05) | 0·36 (0·31, 0·42) | 1·21 (1·10, 1·31) | 1·46 (1·36, 1·57) | 1·87 (1·75, 1·99) | 2·79 (2·64, 2·94) | 4·23 (4·05, 4·41) |
| T3 (most advantaged) | 0·27 (0·23, 0·31) | 2·65 (2·47, 2·84) | 4·67 (4·45, 4·89) | 7·94 (7·64, 8·24) | 9·21 (8·88, 9·55) | 12·73 (12·34, 13·11) | 11·69 (11·32, 12·06) | 0·10 (0·07, 0·12) | 0·92 (0·81, 1·03) | 1·38 (1·26, 1·51) | 2·35 (2·18, 2·51) | 2·72 (2·53, 2·91) | 4·11 (3·88, 4·34) | 3·81 (3·59, 4·03) |
| T3-T1 difference | 0·18 (0·13, 0·23) | 1·92 (1·71, 2·13) | 2·81 (2·54, 3·09) | 5·42 (5·08, 5·77) | 4·86 (4·45, 5·27) | 5·56 (5·07, 6·05) | -0·49 (-1·02, 0·04) | 0·08 (0·05, 0·10) | 0·72 (0·59, 0·84) | 0·93 (0·78, 1·08) | 1·75 (1·56, 1·94) | 1·81 (1·59, 2·03) | 1·90 (1·62, 2·19) | -0·68 (-1·00, -0·35) |
| **Thinness** | | | | | | | | | | | | | | |
| **Urban-rural location** | | | | | | | | | | | | | | |

| | | | | | | | | | | | | | | |
|---|---|---|---|---|---|---|---|---|---|---|---|---|---|---|
| Urban | 11·15 (10·96, 11·34) | 7·70 (7·47, 7·93) | 6·68 (6·46, 6·89) | 5·79 (5·59, 5·98) | 5·09 (4·90, 5·27) | 4·08 (3·91, 4·25) | 3·37 (3·22, 3·52) | 9·60 (9·42, 9·78) | 7·08 (6·86, 7·30) | 6·03 (5·82, 6·23) | 5·17 (4·99, 5·35) | 4·67 (4·49, 4·85) | 3·79 (3·63, 3·96) | 2·95 (2·81, 3·10) |
| Rural | 7·44 (7·28, 7·60) | 8·38 (8·14, 8·62) | 8·63 (8·39, 8·87) | 7·90 (7·68, 8·13) | 6·28 (6·07, 6·49) | 4·84 (4·66, 5·03) | 4·06 (3·90, 4·23) | 5·79 (5·64, 5·93) | 6·60 (6·39, 6·82) | 7·16 (6·94, 7·38) | 6·33 (6·13, 6·53) | 5·29 (5·10, 5·49) | 3·92 (3·75, 4·09) | 3·09 (2·95, 3·24) |
| Urban -rural difference | 3·71 (3·46, 3·96) | -0·68 (-1·01, -0·35) | -1·95 (-2·28, -1·63) | -2·12 (-2·41, -1·82) | -1·19 (-1·47, -0·91) | -0·76 (-1·01, -0·51) | -0·69 (-0·92, -0·46) | 3·81 (3·58, 4·05) | 0·48 (0·17, 0·79) | -1·13 (-1·43, -0·83) | -1·16 (-1·43, -0·89) | -0·63 (-0·89, -0·36) | -0·13 (-0·36, 0·11) | -0·14 (-0·34, 0·07) |
| **Regional socioeconomic status** | | | | | | | | | | | | | | |
| T1 (most disadvantaged) | 9·25 (9·00, 9·50) | 9·68 (9·35, 10·01) | 9·61 (9·27, 9·95) | 9·49 (9·16, 9·82) | 7·47 (7·16, 7·77) | 5·48 (5·21, 5·74) | 4·10 (3·87, 4·33) | 7·58 (7·35, 7·81) | 8·00 (7·69, 8·31) | 7·58 (7·27, 7·88) | 7·25 (6·96, 7·54) | 5·95 (5·68, 6·23) | 4·15 (3·92, 4·38) | 3·42 (3·21, 3·63) |
| T2 | 9·82 (9·63, 10·01) | 8·30 (8·04, 8·57) | 7·54 (7·29, 7·79) | 6·54 (6·33, 6·76) | 5·44 (5·24, 5·65) | 4·51 (4·32, 4·70) | 3·53 (3·37, 3·70) | 8·19 (8·01, 8·36) | 6·93 (6·69, 7·17) | 6·72 (6·48, 6·95) | 5·54 (5·34, 5·74) | 4·98 (4·78, 5·17) | 4·00 (3·82, 4·18) | 2·63 (2·49, 2·77) |
| T3 (most advantaged) | 8·47 (8·24, 8·69) | 5·90 (5·63, 6·17) | 6·15 (5·90, 6·40) | 4·68 (4·44, 4·91) | 4·29 (4·06, 4·52) | 3·37 (3·16, 3·58) | 3·65 (3·43, 3·86) | 6·98 (6·78, 7·19) | 5·55 (5·29, 5·81) | 5·61 (5·37, 5·86) | 4·58 (4·34, 4·81) | 4·03 (3·80, 4·25) | 3·34 (3·13, 3·55) | 3·28 (3·08, 3·49) |
| T3-T1 difference | -0·78 (-1·12, -0·45) | -3·78 (-4·21, -3·36) | -3·46 (-3·89, -3·04) | -4·82 (-5·22, -4·41) | -3·18 (-3·56, -2·79) | -2·11 (-2·44, -1·77) | -0·46 (-0·77, -0·14) | -0·59 (-0·90, -0·29) | -2·45 (-2·85, -2·04) | -1·96 (-2·35, -1·57) | -2·68 (-3·05, -2·31) | -1·93 (-2·28, -1·57) | -0·81 (-1·12, -0·50) | -0·14 (-0·43, 0·15) |

## Table S4. Urban-rural difference in prevalence of obesity and thinness from 1985 to 2019, by regional socioeconomic status for boys and girls

| | Boys | | | | | | | Girls | | | | | | |
|---|---|---|---|---|---|---|---|---|---|---|---|---|---|---|
| | 1985 | 1995 | 2000 | 2005 | 2010 | 2014 | 2019 | 1985 | 1995 | 2000 | 2005 | 2010 | 2014 | 2019 |
| **Obesity** | | | | | | | | | | | | | | |
| **T1 (most disadvantaged)** | | | | | | | | | | | | | | |
| Urban | 0·11 (0·07, 0·15) | 1·23 (1·05, 1·40) | 3·03 (2·75, 3·31) | 3·86 (3·55, 4·16) | 6·32 (5·92, 6·71) | 9·22 (8·74, 9·69) | 13·10 (12·55, 13·66) | 0·02 (0·00, 0·03) | 0·33 (0·24, 0·42) | 0·77 (0·63, 0·91) | 0·80 (0·65, 0·94) | 1·31 (1·13, 1·50) | 2·64 (2·38, 2·90) | 4·87 (4·52, 5·22) |
| Rural | 0·06 (0·03, 0·09) | 0·23 (0·15, 0·30) | 0·66 (0·53, 0·80) | 1·20 (1·03, 1·37) | 2·39 (2·14, 2·64) | 5·12 (4·76, 5·48) | 11·23 (10·71, 11·76) | 0·03 (0·01, 0·05) | 0·06 (0·02, 0·11) | 0·13 (0·07, 0·19) | 0·40 (0·30, 0·50) | 0·51 (0·39, 0·63) | 1·77 (1·56, 1·99) | 4·11 (3·78, 4·44) |
| Urban-rural difference | 0·05 (0·00, 0·10) | 1·00 (0·81, 1·19) | 2·37 (2·06, 2·68) | 2·66 (2·31, 3·01) | 3·92 (3·45, 4·39) | 4·10 (3·50, 4·69) | 1·87 (1·11, 2·63) | -0·01 (-0·04, 0·01) | 0·27 (0·17, 0·37) | 0·64 (0·48, 0·79) | 0·40 (0·23, 0·57) | 0·80 (0·58, 1·02) | 0·87 (0·53, 1·21) | 0·76 (0·28, 1·24) |
| **T2** | | | | | | | | | | | | | | |
| Urban | 0·16 (0·13, 0·20) | 2·00 (1·81, 2·18) | 5·16 (4·86, 5·45) | 7·54 (7·22, 7·86) | 9·29 (8·92, 9·66) | 11·11 (10·71, 11·52) | 14·40 (13·96, 14·84) | 0·06 (0·04, 0·08) | 0·58 (0·48, 0·69) | 1·54 (1·38, 1·71) | 2·05 (1·88, 2·23) | 2·37 (2·17, 2·56) | 3·28 (3·05, 3·51) | 4·57 (4·30, 4·83) |
| Rural | 0·04 (0·03, 0·06) | 0·39 (0·31, 0·48) | 2·02 (1·83, 2·22) | 2·90 (2·70, 3·11) | 5·02 (4·73, 5·30) | 7·87 (7·52, 8·21) | 11·01 (10·61, 11·40) | 0·02 (0·01, 0·04) | 0·14 (0·09, 0·19) | 0·86 (0·73, 0·98) | 0·84 (0·73, 0·96) | 1·37 (1·22, 1·52) | 2·29 (2·10, 2·48) | 3·90 (3·66, 4·15) |
| Urban-rural difference | 0·12 (0·08, 0·16) | 1·60 (1·40, 1·81) | 3·13 (2·78, 3·49) | 4·64 (4·26, 5·02) | 4·28 (3·81, 4·74) | 3·24 (2·71, 3·78) | 3·39 (2·80, 3·98) | 0·03 (0·01, 0·06) | 0·44 (0·33, 0·56) | 0·69 (0·48, 0·89) | 1·21 (1·00, 1·42) | 0·99 (0·75, 1·24) | 0·99 (0·69, 1·29) | 0·66 (0·30, 1·02) |
| **T3 (most advantaged)** | | | | | | | | | | | | | | |
| Urban | 0·48 (0·41, 0·56) | 4·03 (3·71, 4·35) | 7·27 (6·87, 7·66) | 10·87 (10·38, 11·36) | 11·42 (10·9, 11·94) | 14·17 (13·60, 14·74) | 12·54 (12·00, 13·07) | 0·17 (0·12, 0·21) | 1·37 (1·18, 1·56) | 2·04 (1·83, 2·26) | 2·99 (2·72, 3·26) | 3·07 (2·79, 3·36) | 4·18 (3·85, 4·51) | 3·63 (3·32, 3·93) |
| Rural | 0·05 (0·03, 0·08) | 1·28 (1·10, 1·47) | 2·24 (2·02, 2·46) | 4·99 (4·65, 5·34) | 7·00 (6·59, 7·42) | 11·29 (10·77, 11·81) | 10·82 (10·32, 11·33) | 0·03 (0·01, 0·05) | 0·48 (0·37, 0·59) | 0·76 (0·63, 0·89) | 1·69 (1·49, 1·89) | 2·36 (2·12, 2·61) | 4·04 (3·72, 4·37) | 3·99 (3·68, 4·31) |
| Urban-rural difference | 0·43 (0·35, 0·52) | 2·75 (2·38, 3·12) | 5·03 (4·58, 5·48) | 5·88 (5·28, 6·48) | 4·42 (3·75, 5·09) | 2·88 (2·10, 3·65) | 1·71 (0·98, 2·45) | 0·14 (0·09, 0·19) | 0·89 (0·67, 1·11) | 1·28 (1·03, 1·53) | 1·30 (0·96, 1·64) | 0·71 (0·34, 1·09) | 0·13 (-0·33, 0·59) | -0·37 (-0·80, -0·07) |
| **Thinness** | | | | | | | | | | | | | | |
| **T1 (most disadvantaged)** | | | | | | | | | | | | | | |
| Urban | 11·97 (11·57, 12·37) | 10·02 (9·55, 10·49) | 9·24 (8·76, 9·71) | 8·08 (7·65, 8·51) | 6·73 (6·32, 7·14) | 4·92 (4·56, 5·27) | 3·92 (3·60, 4·24) | 9·96 (9·59, 10·32) | 8·50 (8·07, 8·93) | 7·26 (6·84, 7·69) | 6·39 (6·00, 6·78) | 5·46 (5·09, 5·84) | 3·91 (3·59, 4·23) | 3·34 (3·04, 3·63) |

| | | | | | | | | | | | | | | |
|---|---|---|---|---|---|---|---|---|---|---|---|---|---|---|
| Rural | 6·53 (6·23, 6·84) | 9·33 (8·87, 9·80) | 10·00 (9·50, 10·49) | 10·88 (10·39, 11·37) | 8·21 (7·76, 8·66) | 6·03 (5·64, 6·42) | 4·29 (3·95, 4·63) | 5·20 (4·93, 5·47) | 7·43 (6·99, 7·87) | 7·89 (7·45, 8·34) | 8·10 (7·67, 8·52) | 6·44 (6·04, 6·84) | 4·38 (4·05, 4·72) | 3·51 (3·20, 3·81) |
| Urban-rural difference | 5·44 (4·94, 5·93) | 0·68 (0·02, 1·34) | -0·76 (-1·45, -0·08) | -2·79 (-3·44, -2·14) | -1·48 (-2·09, -0·87) | -1·12 (-1·64, -0·59) | -0·37 (-0·72, -0·08) | 4·76 (4·31, 5·22) | 1·07 (0·46, 1·69) | -0·63 (-1·24, -0·01) | -1·70 (-2·28, -1·12) | -0·97 (-1·52, -0·42) | -0·47 (-0·93, -0·01) | -0·17 (-0·59, 0·26) |
| **T2** | | | | | | | | | | | | | | |
| Urban | 11·57 (11·28, 11·86) | 7·56 (7·21, 7·91) | 6·60 (6·27, 6·94) | 5·53 (5·25, 5·80) | 4·73 (4·46, 5·01) | 4·03 (3·78, 4·28) | 3·15 (2·93, 3·37) | 9·92 (9·65, 10·19) | 7·02 (6·68, 7·36) | 6·02 (5·70, 6·34) | 5·03 (4·76, 5·29) | 4·58 (4·32, 4·85) | 3·97 (3·72, 4·22) | 2·50 (2·30, 2·69) |
| Rural | 8·08 (7·83, 8·32) | 9·05 (8·67, 9·43) | 8·53 (8·15, 8·92) | 7·59 (7·26, 7·92) | 6·16 (5·85, 6·46) | 4·99 (4·71, 5·27) | 3·92 (3·68, 4·17) | 6·46 (6·24, 6·68) | 6·85 (6·51, 7·18) | 7·45 (7·09, 7·81) | 6·07 (5·77, 6·37) | 5·37 (5·08, 5·66) | 4·03 (3·77, 4·28) | 2·76 (2·56, 2·97) |
| Urban-rural difference | 3·49 (3·11, 3·87) | -1·49 (-2·01, -0·97) | -1·93 (-2·44, -1·42) | -2·07 (-2·49, -1·64) | -1·42 (-1·83, -1·01) | -0·96 (-1·33, -0·58) | -0·77 (-1·10, -0·45) | 3·47 (3·12, 3·81) | 0·17 (-0·31, 0·65) | -1·43 (-1·91, -0·95) | -1·05 (-1·44, -0·65) | -0·78 (-1·18, -0·39) | -0·05 (-0·41, 0·30) | -0·27 (-0·55, 0·02) |
| **T3 (most advantaged)** | | | | | | | | | | | | | | |
| Urban | 9·74 (9·40, 10·08) | 5·39 (5·02, 5·75) | 4·55 (4·23, 4·87) | 3·95 (3·64, 4·25) | 4·02 (3·70, 4·34) | 3·32 (3·03, 3·62) | 3·21 (2·93, 3·50) | 8·76 (8·44, 9·09) | 5·64 (5·26, 6·01) | 4·96 (4·63, 5·29) | 4·21 (3·89, 4·52) | 4·01 (3·69, 4·33) | 3·39 (3·09, 3·69) | 3·33 (3·04, 3·62) |
| Rural | 7·19 (6·89, 7·49) | 6·41 (6·01, 6·80) | 7·65 (7·26, 8·04) | 5·41 (5·05, 5·77) | 4·56 (4·22, 4·90) | 3·42 (3·12, 3·71) | 4·09 (3·77, 4·41) | 5·21 (4·95, 5·46) | 5·46 (5·09, 5·83) | 6·23 (5·88, 6·59) | 4·95 (4·61, 5·30) | 4·04 (3·72, 4·36) | 3·29 (2·99, 3·58) | 3·23 (2·95, 3·52) |
| Urban-rural difference | 2·55 (2·10, 3·00) | -1·02 (-1·56, -0·48) | -3·10 (-3·61, -2·60) | -1·46 (-1·93, -0·99) | -0·53 (-1·00, -0·07) | -0·10 (-0·51, 0·32) | -0·87 (-1·30, -0·44) | 3·55 (3·14, 3·97) | 0·18 (-0·35, 0·70) | -1·27 (-1·76, -0·79) | -0·75 (-1·22, -0·28) | -0·03 (-0·48, 0·43) | 0·10 (-0·31, 0·52) | 0·10 (-0·31, 0·51) |

**Table S5. The prevalence of obesity and thinness, and annual change, by regional socioeconomic status strata, 1985-2019**

| | 1985 | 1995 | 2000 | 2005 | 2010 | 2014 | 2019 |
|---|---|---|---|---|---|---|---|
| **Obesity prevalence (95% CIs)** | | | | | | | |
| T1 (most disadvantaged) | 0·05 (0·04, 0·07) | 0·48 (0·42, 0·53) * | 1·16 (1·07, 1·24) * | 1·56 (1·46, 1·66) * | 2·63 (2·50, 2·76) * | 4·69 (4·51, 4·86) * | 8·33 (8·10, 8·56) * |
| T2 | 0·07 (0·06, 0·08) | 0·78 (0·72, 0·84) * | 2·42 (2·32, 2·53) * | 3·37 (3·26, 3·48) * | 4·51 (4·38, 4·64) * | 6·14 (5·99, 6·30) * | 8·52 (8·34, 8·69) * |
| T3 (most advantaged) | 0·18 (0·16, 0·21) | 1·79 (1·68, 1·90) * | 3·03 (2·90, 3·16) * | 5·15 (4·98, 5·32) * | 5·97 (5·77, 6·16) * | 8·42 (8·19, 8·65) * | 7·73 (7·52, 7·95) * |
| T3-T1 difference | 0·13 (0·10, 0·16) | 1·31 (1·19, 1·43) | 1·88 (1·72, 2·03) | 3·59 (3·39, 3·79) | 3·33 (3·10, 3·57) | 3·73 (3·45, 4·02) | -0·60 (-0·91, -0·29) |
| **Thinness prevalence (95% CIs)** | | | | | | | |
| T1 (most disadvantaged) | 8·42 (8·25, 8·59) | 8·85 (8·63, 9·08) * | 8·59 (8·36, 8·82) | 8·38 (8·16, 8·59) | 6·71 (6·51, 6·92) * | 4·81 (4·64, 4·99) * | 3·76 (3·60, 3·92) * |
| T2 | 9·00 (8·88, 9·13) | 7·62 (7·44, 7·80) * | 7·13 (6·95, 7·30) * | 6·04 (5·89, 6·19) * | 5·21 (5·07, 5·35) * | 4·25 (4·12, 4·38) * | 3·09 (2·98, 3·20) * |
| T3 (most advantaged) | 7·73 (7·57, 7·88) | 5·72 (5·54, 5·91) * | 5·88 (5·71, 6·06) | 4·63 (4·46, 4·79) * | 4·16 (4·00, 4·32) * | 3·35 (3·20, 3·50) * | 3·46 (3·32, 3·61) |
| T3-T1 difference | -0·69 (-0·92, -0·46) | -3·13 (-3·42, -2·84) | -2·71 (-3·00, -2·42) | -3·75 (-4·02, -3·47) | -2·55 (-2·81, -2·29) | -1·46 (-1·69, -1·23) | -0·30 (-0·51, -0·08) |
| | | **1985-1995** | **1995-2000** | **2000-2005** | **2005-2010** | **2010-2014** | **2014-2019** |
| **Annual percentage change in obesity prevalence (95% CIs)** | | | | | | | |
| T1 (most disadvantaged) | - | 0·04 (0·04, 0·05) | 0·14 (0·11, 0·16) | 0·08 (0·05, 0·11) | 0·21 (0·18, 0·25) | 0·51 (0·46, 0·57) | 0·73 (0·67, 0·79) |
| T2 | - | 0·07 (0·06, 0·08) | 0·33 (0·30, 0·35) | 0·19 (0·16, 0·22) | 0·23 (0·19, 0·26) | 0·41 (0·36, 0·46) | 0·47 (0·43, 0·52) |
| T3 (most advantaged) | - | 0·16 (0·15, 0·17) | 0·25 (0·21, 0·28) | 0·42 (0·38, 0·47) | 0·16 (0·11, 0·22) | 0·61 (0·54, 0·69) | -0·14 (-0·20, -0·07) |
| **Annual percentage change in thinness prevalence (95% CIs)** | | | | | | | |
| T1 (most disadvantaged) | - | 0·04 (0·02, 0·07) | -0·05 (-0·12, 0·01) | -0·04 (-0·11, 0·02) | -0·33 (-0·39, -0·27) | -0·48 (-0·54, -0·41) | -0·21 (-0·26, -0·16) |
| T2 | - | -0·14 (-0·16, -0·12) | -0·10 (-0·15, -0·05) | -0·22 (-0·26, -0·17) | -0·17 (-0·21, -0·13) | -0·24 (-0·29, -0·19) | -0·23 (-0·27, -0·20) |
| T3 (most advantaged) | - | -0·20 (-0·22, -0·18) | 0·03 (-0·02, 0·08) | -0·25 (-0·30, -0·20) | -0·09 (-0·14, -0·05) | -0·20 (-0·26, -0·15) | 0·02 (-0·02, 0·06) |

*Note*: Difference of prevalences between two adjacent surveys was examined by $\chi^2$ test, $^{*}p < 0.05$.

**Table S6. Projected prevalence of obesity and thinness in 2025 and 2030, by urban-rural location and sex.**

| | Boys | | Girls | |
|---|---|---|---|---|
| | 2025 | 2030 | 2025 | 2030 |
| **Obesity** | | | | |
| Urban | 17.93(17.48,18.28) | 21.88(21.3,22.57) | 5.97(4.12,6.34) | 7.76(4.5,8.31) |
| Rural | 18.45(13.37,18.9) | 25.92(16.46,26.95) | 6.98(4.92,7.37) | 10.64(4.32,11.42) |
| Urban-rural difference | -0.52(-3.32,2.27) | -4.04(-9.32,1.24) | -1(-2.65,0.65) | -2.88(-6.91,1.15) |
| **Thinness** | | | | |
| Urban | 2.65(2.54,2.78) | 2.1(1.99,2.22) | 2.42(2.32,2.57) | 1.95(1.82,2.05) |
| Rural | 2.77(2.55,3.11) | 1.92(1.77,2.26) | 2.28(2.18,2.38) | 1.65(1.56,1.75) |
| Urban-rural difference | -0.12(-0.42,0.19) | 0.14(-0.02,0.31) | 0.18(-0.09,0.46) | 0.3(0.15,0.44) |

**Table S7. Projected prevalence of obesity and thinness in 2025 and 2030, by regional SES and sex.**

| | Boys | | Girls | |
|---|---|---|---|---|
| | 2025 | 2030 | 2025 | 2030 |
| **Obesity** | | | | |
| T1 | 20.17(19.55,20.88) | 29.32(28.09,30.63) | 9.18(8.44,9.89) | 16.05(14.06,17.92) |
| T3 | 17(16.46,17.57) | 20.43(19.58,21.4) | 5.77(5.41,6.08) | 7.23(6.73,7.79) |
| T3-T1 difference | -3.17(-5.88,-0.47) | -8.88(-13.76,-4.01) | -3.41(-5.17,-1.66) | -8.82(-12.78,-4.85) |
| **Thinness** | | | | |
| T1 | 2.97(2.8,3.09) | 2.47(2.31,2.62) | 2(1.86,2.15) | 1.71(1.59,1.85) |
| T3 | 2.64(2.51,2.75) | 2.48(2.33,2.68) | 2.18(2.04,2.32) | 2.06(1.9,2.23) |
| T3-T1 difference | -0.33(-0.63,-0.03) | 0.18(-0.11,0.47) | 0(-0.16,0.17) | 0.36(0.21,0.51) |

**Table S8. Characteristics of ethnic minority participants from four national surveys of CNSSCH from 2005 to 2019**

| Variables | **1985** | **1995** | **2000** | **2005** (n = 48400) | **2010** (n = 41999) | **2014** (n = 46741) | **2019** (n = 68338) |
|---|---|---|---|---|---|---|---|
| **Sex, n (%)** | | | | | | | |
| Boys | / | / | / | 24180(50·0) | 21137(50·3) | 23322(49·9) | 34216(50·1) |
| Girls | / | / | / | 24220(50·0) | 20862(49·7) | 23419(50·1) | 34122(49·9) |
| **Age (years), n (%)** | | | | | | | |
| 7-9 | / | / | / | 11903(24·6) | 10530(25·1) | 11636(24·9) | 17562(25·7) |
| 10-12 | / | / | / | 12227(25·3) | 10439(24·9) | 11661(24·9) | 17547(25·7) |
| 13-15 | / | / | / | 12232(25·3) | 10591(25·2) | 11906(25·5) | 17203(25·2) |
| 16-18 | / | / | / | 12038(24·9) | 10439(24·9) | 11538(24·7) | 16026(23·5) |
| **Urban-rural residence, n (%)** | | | | | | | |
| Urban | / | / | / | 17509(36·2) | 20718(49·3) | 22038(47·1) | 24414(35·7) |
| Rural | / | / | / | 30891(63·8) | 21281(50·7) | 24703(52·9) | 43924(64·3) |
| **Regional SES, n (%)** | | | | | | | |
| T1 (most disadvantaged) | / | / | / | 15057(32·7) | 9591(24·4) | 10117(22·8) | 23009(39·4) |
| T2 | / | / | / | 30946(67·3) | 25932(65·9) | 29099(65·7) | 35380(60·6) |
| T3 (most advantaged) | / | / | / | / | 3834(9·7) | 5107(11·5) | / |

Note: GDP = Gross Domestic Product; SES = socioeconomic status

**Table S9. The prevalence of obesity and thinness among ethnic minority participants aged 7-18, by regional socioeconomic status, 2005-2019**

| **Boys** | **1985** | **1995** | **2000** | **2005** | **2010** | **2014** | **2019** |
|---|---|---|---|---|---|---|---|
| **Obesity prevalence (95% CIs)** | | | | | | | |
| T1 | / | / | / | 1.43 (1.16 ,1.70) | 1.84 (1.46 ,2.22) | 5.07 (4.47 ,5.68) | 8.01 (7.52 ,8.51) |
| T2 | / | / | / | 2.53 (2.29 ,2.78) | 4.84 (4.47 ,5.21) | 6.27 (5.88 ,6.67) | 7.52 (7.13 ,7.91) |
| T1-T2 difference | / | / | / | 1.10 (0.74 ,1.47) | 3.00 (2.47 ,3.52) | 1.20 (0.48 ,1.92) | -0.49 (-1.13 ,0.14) |
| **Thinness prevalence (95% CIs)** | | | | | | | |
| T1 | / | / | / | 9.47(8.81,10.14) | 12.48(11.54,13.42) | 6.4(5.72,7.08) | 3.65(3.31,4) |
| T2 | / | / | / | 3.82(3.52,4.13) | 4.81(4.44,5.17) | 4.24(3.91,4.57) | 3.16(2.91,3.42) |
| T1-T2 difference | / | / | / | -5.65(-6.38,-4.92) | -7.67(-8.68,-6.67) | -2.16(-2.91,-1.41) | -0.49(-0.92,-0.06) |
| **Girls** | **1985** | **1995** | **2000** | **2005** | **2010** | **2014** | **2019** |
| **Obesity prevalence (95% CIs)** | | | | | | | |
| T1 | / | / | / | 0.20 (0.10 ,0.30) | 0.66 (0.43 ,0.89) | 1.54 (1.20 ,1.88) | 3.14 (2.82 ,3.46) |
| T2 | / | / | / | 1.10 (0.94 ,1.27) | 1.93 (1.69 ,2.17) | 2.35 (2.10 ,2.59) | 3.18 (2.93 ,3.44) |
| T1-T2 difference | / | / | / | 0.90 (0.71 ,1.10) | 1.26 (0.93 ,1.59) | 0.81 (0.39 ,1.23) | 0.05 (-0.36 ,0.46) |
| **Thinness prevalence (95% CIs)** | | | | | | | |
| T1 | / | / | / | 8.02(7.41,8.63) | 10.51(9.64,11.37) | 7.18(6.47,7.89) | 2.82(2.51,3.12) |
| T2 | / | / | / | 3.48(3.2,3.77) | 4.5(4.14,4.86) | 4(3.68,4.32) | 2.21(1.99,2.43) |
| T1-T2 difference | / | / | / | -4.53(-5.21, -3.86) | -6.01(-6.95, -5.07) | -3.18(-3.96, -2.4) | -0.61(-0.98, -0.23) |

*Note*: Difference of prevalences between two adjacent surveys was examined by $\chi^2$ test, $^{*}P < 0.05$.

**Table S10. The prevalence of obesity and thinness among ethnic minority participants aged 7-18, by urban-rural location, 2005-2019**

| Boys | 1985 | 1995 | 2000 | 2005 | 2010 | 2014 | 2019 |
|---|---|---|---|---|---|---|---|
| **Obesity prevalence (95% CIs)** | | | | | | | |
| Urban | / | / | / | 3.42(3.04,3.8) | 5.35(4.92,5.78) | 8.05(7.55,8.56) | 8.97(8.46,9.47) |
| Rural | / | / | / | 1.42(1.23,1.6) | 3.06(2.73,3.38) | 4.73(4.35,5.1) | 5.95(5.64,6.26) |
| Urban-rural difference | / | / | / | 2.01(1.58,2.43) | 2.29(1.75,2.83) | 3.33(2.7,3.96) | 3.02(2.42,3.61) |
| **Thinness prevalence (95% CIs)** | | | | | | | |
| Urban | / | / | / | 5.35(4.88,5.82) | 6.05(5.59,6.5) | 4.05(3.68,4.42) | 2.87(2.57,3.16) |
| Rural | / | / | / | 5.87(5.5,6.24) | 7.1(6.61,7.59) | 4.9(4.52,5.28) | 3.67(3.42,3.92) |
| Urban-rural difference | / | / | / | -0.52(-1.12,0.08) | -1.05(-1.72,-0.38) | -0.85(-1.38,-0.32) | -0.8(-1.19,-0.42) |
| **Girls** | **1985** | **1995** | **2000** | **2005** | **2010** | **2014** | **2019** |
| **Obesity prevalence (95% CIs)** | | | | | | | |
| Urban | / | / | / | 1.15(0.92,1.37) | 1.9(1.64,2.17) | 3.06(2.74,3.39) | 3.6(3.27,3.94) |
| Rural | / | / | / | 0.63(0.5,0.75) | 1.39(1.16,1.61) | 1.71(1.48,1.94) | 2.51(2.3,2.71) |
| Urban-rural difference | / | / | / | 0.52(0.27,0.78) | 0.52(0.17,0.86) | 1.35(0.96,1.75) | 1.1(0.71,1.49) |
| **Thinness prevalence (95% CIs)** | | | | | | | |
| Urban | / | / | / | 5.03(4.58,5.49) | 5.6(5.15,6.04) | 4.1(3.73,4.47) | 1.83(1.6,2.07) |
| Rural | / | / | / | 4.92(4.58,5.26) | 6.21(5.75,6.67) | 4.66(4.29,5.03) | 2.72(2.51,2.94) |
| Urban-rural difference | / | / | / | 0.12(-0.45,0.69) | -0.61(-1.25,0.03) | -0.56(-1.08,-0.03) | -0.89(-1.21,-0.57) |

*Note*: Difference of prevalences between two adjacent surveys was examined by $\chi^2$ test, $^*P < 0.05$.

**Table S11. The prevalence of obesity and thinness among participants of Han ethnic, by regional socioeconomic status, 1985-2019**

| Boys | 1985 | 1995 | 2000 | 2005 | 2010 | 2014 | 2019 |
|---|---|---|---|---|---|---|---|
| **Obesity prevalence (95% CIs)** | | | | | | | |
| T1 (most disadvantaged) | 0.3(0.2,0.3) | 2.7(2.5,2.9) | 4.6(4.4,4.8) | 7.7(7.4,8) | 9.4(9,9.7) | 11.9(11.5,12.3) | 11.8(11.4,12.1) |
| T2 | 0.1(0.1,0.1) | 1.3(1.2,1.4) | 3.6(3.4,3.8) | 5.1(4.9,5.2) | 6.5(6.3,6.7) | 9.9(9.6,10.1) | 12.9(12.6,13.2) |
| T3 (most advantaged) | 0.1(0.1,0.1) | 0.6(0.5,0.7) | 2.1(1.9,2.2) | 3.2(3,3.4) | 5.6(5.3,5.8) | 7.6(7.3,8.0) | 11.7(11.3,12.1) |
| T3-T1 difference | 0.2(0.1,0.2) | 2.1(1.9,2.4) | 2.5(2.3,2.8) | 4.5(4.2,4.9) | 3.8(3.4,4.3) | 4.2(3.7,4.8) | 0.1(-0.5,0.6) |
| **Thinness prevalence (95% CIs)** | | | | | | | |
| T1 (most disadvantaged) | 9.6(9.4,9.9) | 5.8(5.5,6.1) | 6.1(5.8,6.3) | 4.8(4.5,5) | 4.3(4.1,4.6) | 3.4(3.2,3.6) | 3.7(3.5,3.9) |
| T2 | 9(8.8,9.2) | 7.8(7.6,8.1) | 7.9(7.7,8.2) | 6.1(5.9,6.4) | 5.6(5.4,5.8) | 4.2(4.1,4.4) | 3.3(3.1,3.5) |
| T3 (most advantaged) | 9.5(9.2,9.8) | 10.3(10,10.7) | 9.1(8.8,9.4) | 9.8(9.5,10.1) | 7.1(6.8,7.4) | 5.8(5.5,6.1) | 4.4(4.2,4.7) |
| T3-T1 difference | 0.1(-0.2,0.5) | -4.5(-5,-4.1) | -3.1(-3.5,-2.6) | -5.1(-5.5,-4.7) | -2.8(-3.2,-2.4) | -2.4(-2.7,-2) | -0.7(-1,-0.4) |
| **Girls** | **1985** | **1995** | **2000** | **2005** | **2010** | **2014** | **2019** |
| **Obesity prevalence (95% CIs)** | | | | | | | |
| T1 (most disadvantaged) | 0.1(0.1,0.1) | 0.9(0.8,1) | 1.5(1.3,1.6) | 2.3(2.1,2.4) | 2.6(2.4,2.8) | 3.7(3.5,3.9) | 3.9(3.7,4.1) |
| T2 | 0(0,0.1) | 0.5(0.4,0.5) | 1(1,1.1) | 1.4(1.3,1.5) | 1.8(1.7,1.9) | 2.9(2.8,3.1) | 4.3(4.1,4.5) |
| T3 (most advantaged) | 0(0,0) | 0.1(0.1,0.2) | 0.6(0.5,0.7) | 0.8(0.7,0.9) | 1.3(1.2,1.4) | 2.5(2.3,2.7) | 4.3(4.1,4.5) |
| T3-T1 difference | 0.1(0.1,0.1) | 0.7(0.6,0.9) | 0.9(0.7,1) | 1.5(1.3,1.7) | 1.3(1,1.5) | 1.2(0.9,1.5) | -0.4(-0.8,-0.1) |
| **Thinness prevalence (95% CIs)** | | | | | | | |
| T1 (most disadvantaged) | 7.9(7.7,8.1) | 5.3(5,5.6) | 5.5(5.3,5.8) | 4.6(4.3,4.8) | 3.9(3.7,4.2) | 3.3(3.1,3.6) | 3.3(3.1,3.5) |
| T2 | 7.6(7.5,7.8) | 6.9(6.6,7.1) | 7(6.8,7.3) | 5.4(5.2,5.6) | 5(4.8,5.1) | 3.6(3.5,3.8) | 2.5(2.3,2.6) |
| T3 (most advantaged) | 7.6(7.4,7.9) | 8.2(7.9,8.5) | 7.2(6.9,7.5) | 7.4(7.2,7.7) | 5.9(5.7,6.2) | 4.7(4.5,5) | 3.6(3.4,3.9) |
| T3-T1 difference | 0.3(-0.1,0.6) | -2.9(-3.3,-2.5) | -1.7(-2.1,-1.3) | -2.9(-3.2,-2.5) | -2(-2.4,-1.6) | -1.4(-1.7,-1) | -0.3(-0.6,0) |

*Note*: Difference of prevalences between two adjacent surveys was examined by $\chi^2$ test, $^*p < 0.05$.

Regional socioeconomic status here was defined using value added of primary sector as per cent of GDP at the provincial level.

**Table S12. The prevalence of obesity and thinness among urban participants of Han ethnic, by regional socioeconomic status, 1985-2019**

| Boys | 1985 | 1995 | 2000 | 2005 | 2010 | 2014 | 2019 |
|---|---|---|---|---|---|---|---|
| **Obesity prevalence (95% CIs)** | | | | | | | |
| T1 (most disadvantaged) | 0.1(0.1,0.1) | 1.2(1.1,1.4) | 3(2.7,3.3) | 3.9(3.6,4.2) | 6.3(5.9,6.7) | 9.2(8.7,9.7) | 13.1(12.5,13.7) |
| T2 | 0.2(0.1,0.2) | 2(1.8,2.2) | 5.2(4.9,5.5) | 7.5(7.2,7.9) | 9.3(8.9,9.7) | 11.1(10.7,11.5) | 14.4(14,14.8) |
| T3 (most advantaged) | 0.5(0.4,0.6) | 4(3.7,4.4) | 7.3(6.9,7.7) | 10.9(10.4,11.4) | 11.4(10.9,11.9) | 14.2(13.6,14.7) | 12.5(12,13.1) |
| T3-T1 difference | 0.4(0.3,0.5) | 2.8(2.4,3.2) | 4.2(3.8,4.7) | 7(6.4,7.6) | 5.1(4.5,5.8) | 5(4.2,5.7) | -0.6(-1.3,0.2) |
| **Thinness prevalence (95% CIs)** | | | | | | | |
| T1 (most disadvantaged) | 12(11.6,12.4) | 10(9.5,10.5) | 9.2(8.8,9.7) | 8.1(7.7,8.5) | 6.7(6.3,7.1) | 4.9(4.6,5.3) | 3.9(3.6,4.2) |
| T2 | 11.6(11.3,11.9) | 7.6(7.2,7.9) | 6.6(6.3,6.9) | 5.5(5.2,5.8) | 4.7(4.5,5) | 4(3.8,4.3) | 3.1(2.9,3.4) |
| T3 (most advantaged) | 9.7(9.4,10.1) | 5.4(5,5.8) | 4.5(4.2,4.9) | 3.9(3.6,4.3) | 4(3.7,4.3) | 3.3(3,3.6) | 3.2(2.9,3.5) |
| T3-T1 difference | -2.2(-2.7,-1.7) | -4.6(-5.2,-4) | -4.7(-5.3,-4.1) | -4.1(-4.7,-3.6) | -2.7(-3.2,-2.2) | -1.6(-2.1,-1.1) | -0.7(-1.1,-0.3) |
| **Girls** | **1985** | **1995** | **2000** | **2005** | **2010** | **2014** | **2019** |
| **Obesity prevalence (95% CIs)** | | | | | | | |
| T1 (most disadvantaged) | 0(0,0) | 0.3(0.2,0.4) | 0.8(0.6,0.9) | 0.8(0.7,0.9) | 1.3(1.1,1.5) | 2.6(2.4,2.9) | 4.9(4.5,5.2) |
| T2 | 0.1(0,0.1) | 0.6(0.5,0.7) | 1.5(1.4,1.7) | 2.1(1.9,2.2) | 2.4(2.2,2.6) | 3.3(3.1,3.5) | 4.6(4.3,4.8) |
| T3 (most advantaged) | 0.2(0.1,0.2) | 1.4(1.2,1.6) | 2(1.8,2.3) | 3(2.7,3.3) | 3.1(2.8,3.4) | 4.2(3.8,4.5) | 3.6(3.3,3.9) |
| T3-T1 difference | 0.2(0.1,0.2) | 1(0.8,1.2) | 1.3(1,1.5) | 2.2(1.9,2.5) | 1.8(1.4,2.1) | 1.5(1.1,2) | -1.2(-1.7,-0.8) |
| **Thinness prevalence (95% CIs)** | | | | | | | |
| T1 (most disadvantaged) | 10(9.6,10.3) | 8.5(8.1,8.9) | 7.3(6.8,7.7) | 6.4(6,6.8) | 5.5(5.1,5.8) | 3.9(3.6,4.2) | 3.3(3,3.6) |
| T2 | 9.9(9.7,10.2) | 7(6.7,7.4) | 6(5.7,6.3) | 5(4.8,5.3) | 4.6(4.3,4.9) | 4(3.7,4.2) | 2.5(2.3,2.7) |
| T3 (most advantaged) | 8.8(8.4,9.1) | 5.6(5.3,6) | 5(4.6,5.3) | 4.2(3.9,4.5) | 4(3.7,4.3) | 3.4(3.1,3.7) | 3.3(3,3.6) |
| T3-T1 difference | -1.2(-1.7,-0.7) | -2.9(-3.4,-2.3) | -2.3(-2.8,-1.8) | -2.2(-2.7,-1.7) | -1.5(-1.9,-1) | -0.5(-1,-0.1) | 0(-0.4,0.4) |

Regional socioeconomic status here was defined using GDP per capita.

**Table S13. The prevalence of obesity and thinness among rural participants of Han ethnic, by regional socioeconomic status, 1985-2019**

| Boys | 1985 | 1995 | 2000 | 2005 | 2010 | 2014 | 2019 |
|---|---|---|---|---|---|---|---|
| **Obesity prevalence (95% CIs)** | | | | | | | |
| T1 (most disadvantaged) | 0.1(0,0.1) | 0.2(0.1,0.3) | 0.7(0.5,0.8) | 1.2(1,1.4) | 2.4(2.1,2.6) | 5.1(4.8,5.5) | 11.2(10.7,11.8) |
| T2 | 0(0,0.1) | 0.4(0.3,0.5) | 2(1.8,2.2) | 2.9(2.7,3.1) | 5(4.7,5.3) | 7.9(7.5,8.2) | 11(10.6,11.4) |
| T3 (most advantaged) | 0.1(0,0.1) | 1.3(1.1,1.5) | 2.2(2,2.5) | 5(4.6,5.3) | 7(6.6,7.4) | 11.3(10.8,11.8) | 10.8(10.3,11.3) |
| T3-T1 difference | 0(-0.1,0) | 1.1(0.9,1.3) | 1.6(1.3,1.8) | 3.8(3.4,4.2) | 4.6(4.1,5.1) | 6.2(5.5,6.8) | -0.4(-1.1,0.3) |
| **Thinness prevalence (95% CIs)** | | | | | | | |
| T1 (most disadvantaged) | 6.5(6.2,6.8) | 9.3(8.9,9.8) | 10(9.5,10.5) | 10.9(10.4,11.4) | 8.2(7.8,8.7) | 6(5.6,6.4) | 4.3(4,4.6) |
| T2 | 8.1(7.8,8.3) | 9(8.7,9.4) | 8.5(8.1,8.9) | 7.6(7.3,7.9) | 6.2(5.8,6.5) | 5(4.7,5.3) | 3.9(3.7,4.2) |
| T3 (most advantaged) | 7.2(6.9,7.5) | 6.4(6,6.8) | 7.7(7.3,8) | 5.4(5.1,5.8) | 4.6(4.2,4.9) | 3.4(3.1,3.7) | 4.1(3.8,4.4) |
| T3-T1 difference | 0.7(0.2,1.1) | -2.9(-3.5,-2.3) | -2.3(-3,-1.7) | -5.5(-6.1,-4.9) | -3.7(-4.2,-3.1) | -2.6(-3.1,-2.1) | -0.2(-0.7,0.3) |
| **Girls** | **1985** | **1995** | **2000** | **2005** | **2010** | **2014** | **2019** |
| **Obesity prevalence (95% CIs)** | | | | | | | |
| T1 (most disadvantaged) | 0(0,0) | 0.1(0,0.1) | 0.1(0.1,0.2) | 0.4(0.3,0.5) | 0.5(0.4,0.6) | 1.8(1.6,2) | 4.1(3.8,4.4) |
| T2 | 0(0,0) | 0.1(0.1,0.2) | 0.9(0.7,1) | 0.8(0.7,1) | 1.4(1.2,1.5) | 2.3(2.1,2.5) | 3.9(3.7,4.1) |
| T3 (most advantaged) | 0(0,0) | 0.5(0.4,0.6) | 0.8(0.6,0.9) | 1.7(1.5,1.9) | 2.4(2.1,2.6) | 4(3.7,4.4) | 4(3.7,4.3) |
| T3-T1 difference | 0(0,0) | 0.4(0.3,0.5) | 0.6(0.5,0.8) | 1.3(1.1,1.5) | 1.9(1.6,2.1) | 2.3(1.9,2.7) | -0.1(-0.6,0.3) |
| **Thinness prevalence (95% CIs)** | | | | | | | |
| T1 (most disadvantaged) | 5.2(4.9,5.5) | 7.4(7,7.9) | 7.9(7.4,8.3) | 8.1(7.7,8.5) | 6.4(6,6.8) | 4.4(4,4.7) | 3.5(3.2,3.8) |
| T2 | 6.5(6.2,6.7) | 6.8(6.5,7.2) | 7.5(7.1,7.8) | 6.1(5.8,6.4) | 5.4(5.1,5.7) | 4(3.8,4.3) | 2.8(2.6,3) |
| T3 (most advantaged) | 5.2(5,5.5) | 5.5(5.1,5.8) | 6.2(5.9,6.6) | 5(4.6,5.3) | 4(3.7,4.4) | 3.3(3,3.6) | 3.2(2.9,3.5) |
| T3-T1 difference | 0(-0.4,0.4) | -2(-2.5,-1.4) | -1.7(-2.2,-1.1) | -3.1(-3.7,-2.6) | -2.4(-2.9,-1.9) | -1.1(-1.5,-0.7) | -0.3(-0.7,0.1) |

Regional socioeconomic status here was defined using GDP per capita.

**Table S14. The formula and parameters of the fit model**

| | | formula | $R^2$ |
|---|---|---|---|
| **Urban-rural gap in obesity** | Boys | y = 1.543+0.0009989×x-9.112e-008×x^2 | 0.8733 |
| | Girls | y = 0.3093+0.0003443×x-3.615e-008×x^2 | 0.9634 |
| **Urban-rural gap in thinness** | Boys | y = -2·7302×exp(-0·0002×x) + 20·6800×exp(-0·0040×x) | 0.9296 |
| | Girls | y = 14·7590×exp(-0·0030×x) - 2·4844×exp(-0·0004×x) | 0.9539 |
| **T3-T1 gap in obesity** | Boys | y = 1.401+0.001418×x-1.409e-007×x^2 | 0.9202 |
| | Girls | y = 0.06013+0.0009559×x-1.008e-007×x^2 | 0.9919 |
| **T3-T1 gap in thinness** | Boys | y = -7·3700×exp(-0·0002×x) + 9·7067×exp(-0·0017×x) | 0.9239 |
| | Girls | y = -6·0710×exp(-0·0003×x) + 6·4892×exp(-0·0011×x) | 0.6881 |

**Table S15. Nine-year compulsory education enrolment rate for school-age children in China, 1990-2020**

| 1990-2005 | | 2006-2020 | |
|---|---|---|---|
| Year | Enrolment Ratio | Year | Enrolment Ratio |
| 1990 | 66.7 | 2006 | 97.0 |
| 1991 | 69.7 | 2007 | 98.0 |
| 1992 | 71.8 | 2008 | 98.5 |
| 1993 | 73.1 | 2009 | 99.9 |
| 1994 | 73.8 | 2010 | 99.9 |
| 1995 | 78.4 | 2011 | 99.9 |
| 1996 | 82.4 | 2012 | 99.9 |
| 1997 | 87.1 | 2013 | 99.9 |
| 1998 | 87.3 | 2014 | 99.9 |
| 1999 | 88.6 | 2015 | 99.9 |
| 2000 | 88.6 | 2016 | 99.9 |
| 2001 | 88.7 | 2017 | 99.9 |
| 2002 | 90.0 | 2018 | 99.9 |
| 2003 | 92.7 | 2019 | 99.9 |
| 2004 | 94.1 | 2020 | 99.9 |
| 2005 | 95.0 | | |

**Table S16. Adjusted prevalence (%) of childhood and adolescent obesity and thinness for boys, by urban-rural location and regional SES**

| | 1985 | 1995 | 2000 | 2005 | 2010 | 2014 | 2019 |
|---|---|---|---|---|---|---|---|
| **Obesity** | | | | | | | |
| Urban | 0.16(0.11,0.23) | 1.56(1.09,2.25) | 4.07(3.16,5.24) | 6.47(5.27,7.92) | 8.23(6.91,9.78) | 10.53(8.93,12.38) | 12.79(11.21,14.55) |
| Rual | 0.03(0.02,0.05) | 0.39(0.27,0.57) | 1.28(0.98,1.67) | 2.55(2.05,3.16) | 4.33(3.6,5.19) | 7.33(6.18,8.68) | 10.33(9.02,11.81) |
| Urban-rural gaps | 0.12(0.06,0.19) | 1.18(0.58,1.78) | 2.79(1.7,3.88) | 3.92(2.49,5.35) | 3.9(2.26,5.54) | 3.2(1.06,5.33) | 2.46(0.28,4.63) |
| | | | | | | | |
| T1 | 0.07(0.03,0.14) | 0.7(0.38,1.3) | 1.72(1.13,2.62) | 2.42(1.83,3.2) | 4.28(3.22,5.68) | 7.04(5.26,9.35) | 11.74(9.09,15.03) |
| T2 | 0.08(0.05,0.13) | 0.88(0.53,1.45) | 2.67(1.92,3.7) | 4.97(4.02,6.13) | 6.43(5.17,7.97) | 8.6(6.88,10.71) | 11.94(9.85,14.4) |
| T3 | 0.18(0.1,0.32) | 1.65(0.89,3.04) | 4.18(2.78,6.23) | 7.08(5.43,9.17) | 8.77(6.69,11.42) | 11.96(9.07,15.6) | 10.8(8.34,13.87) |
| T3-T1 gaps | 0.11(-0.01,0.23) | 0.95(-0.22,2.12) | 2.46(0.57,4.34) | 4.65(2.66,6.65) | 4.49(1.82,7.15) | 4.92(1.07,8.77) | -0.94(-5,3.12) |
| **Thinness** | | | | | | | |
| Urban | 10.76(9.67,11.95) | 7.32(6.5,8.23) | 6.46(5.56,7.49) | 5.1(4.27,6.08) | 4.78(4.16,5.49) | 3.76(3.21,4.39) | 3.13(2.69,3.63) |
| Rual | 7.14(6.39,7.97) | 8.05(7.16,9.04) | 8.52(7.36,9.84) | 7.16(6.02,8.5) | 5.91(5.16,6.77) | 4.47(3.83,5.22) | 3.79(3.27,4.38) |
| Urban-rural gaps | 3.62(2.23,5.01) | -0.73(-2.01,0.55) | -2.06(-3.63,-0.49) | -2.07(-3.6,-0.53) | -1.13(-2.18,-0.09) | -0.71(-1.63,0.2) | -0.66(-1.39,0.07) |
| | | | | | | | |
| T1 | 9.05(7.32,11.14) | 9.61(8.12,11.33) | 9.45(7.41,11.98) | 9.14(7.05,11.78) | 7.28(5.94,8.91) | 5.31(4.11,6.83) | 3.82(2.89,5.02) |
| T2 | 9.35(8.01,10.89) | 7.9(6.86,9.08) | 7.47(6.15,9.05) | 5.82(4.71,7.18) | 5.19(4.41,6.12) | 4.14(3.37,5.07) | 3.29(2.66,4.05) |
| T3 | 8.24(6.75,10.03) | 5.85(4.9,6.97) | 5.91(4.59,7.58) | 4.41(3.35,5.78) | 4.09(3.3,5.05) | 3.14(2.41,4.09) | 3.41(2.58,4.49) |
| T3-T1 gaps | -0.8(-3.32,1.72) | -3.75(-5.66,-1.84) | -3.54(-6.27,-0.81) | -4.73(-7.39,-2.07) | -3.2(-4.92,-1.47) | -2.16(-3.76,-0.56) | -0.41(-1.84,1.02) |

**Table S17. Adjusted prevalence (%) of childhood and adolescent obesity and thinness for girls, by urban-rural location and regional SES**

| | 1985 | 1995 | 2000 | 2005 | 2010 | 2014 | 2019 |
|---|---|---|---|---|---|---|---|
| **Obesity** | | | | | | | |
| Urban | 0.05(0.03,0.08) | 0.44(0.29,0.67) | 1.04(0.77,1.42) | 1.49(1.13,1.95) | 1.88(1.49,2.39) | 2.93(2.4,3.58) | 3.89(3.25,4.64) |
| Rual | 0.02(0.01,0.03) | 0.13(0.08,0.2) | 0.43(0.32,0.6) | 0.71(0.54,0.95) | 1.16(0.91,1.48) | 2.29(1.87,2.81) | 3.55(2.97,4.25) |
| Urban-rural gaps | 0.04(0.01,0.06) | 0.31(0.12,0.51) | 0.61(0.26,0.96) | 0.77(0.31,1.23) | 0.72(0.19,1.26) | 0.64(-0.12,1.39) | 0.33(-0.61,1.28) |
| T1 | 0.02(0.01,0.04) | 0.17(0.08,0.33) | 0.41(0.25,0.68) | 0.56(0.37,0.86) | 0.86(0.6,1.24) | 2.16(1.53,3.06) | 4.16(2.97,5.78) |
| T2 | 0.03(0.02,0.05) | 0.26(0.15,0.45) | 0.81(0.55,1.2) | 1.23(0.89,1.7) | 1.61(1.22,2.12) | 2.36(1.8,3.09) | 3.82(2.96,4.91) |
| T3 | 0.08(0.04,0.13) | 0.63(0.33,1.19) | 1.17(0.73,1.89) | 1.8(1.2,2.71) | 2.45(1.73,3.46) | 3.71(2.64,5.2) | 3.17(2.26,4.44) |
| T3-T1 gaps | 0.06(0.01,0.11) | 0.46(0.01,0.9) | 0.76(0.14,1.38) | 1.24(0.44,2.04) | 1.59(0.67,2.51) | 1.55(0.06,3.04) | -0.98(-2.76,0.79) |
| **Thinness** | | | | | | | |
| Urban | 9.29(8.4,10.26) | 6.82(6.14,7.57) | 5.9(5.2,6.68) | 4.81(4.16,5.56) | 4.44(3.92,5.03) | 3.54(3.06,4.08) | 2.74(2.36,3.19) |
| Rual | 5.57(5.01,6.19) | 6.45(5.8,7.16) | 7.12(6.29,8.04) | 5.98(5.19,6.89) | 5.04(4.45,5.7) | 3.66(3.17,4.22) | 2.89(2.49,3.35) |
| Urban-rural gaps | 3.72(2.61,4.82) | 0.37(-0.62,1.36) | -1.22(-2.37,-0.07) | -1.17(-2.27,-0.07) | -0.6(-1.43,0.24) | -0.12(-0.85,0.61) | -0.15(-0.74,0.45) |
| T1 | 7.45(6.1,9.06) | 7.93(6.73,9.33) | 7.39(5.96,9.12) | 6.92(5.47,8.73) | 5.8(4.73,7.08) | 4.05(3.13,5.22) | 3.26(2.49,4.25) |
| T2 | 7.81(6.75,9.01) | 6.67(5.82,7.64) | 6.68(5.64,7.91) | 5.29(4.38,6.37) | 4.76(4.05,5.58) | 3.65(2.98,4.46) | 2.48(2.02,3.04) |
| T3 | 6.83(5.66,8.21) | 5.5(4.63,6.51) | 5.48(4.4,6.81) | 4.33(3.39,5.5) | 3.85(3.12,4.74) | 3.11(2.4,4.03) | 3.03(2.32,3.96) |
| T3-T1 gaps | -0.62(-2.58,1.33) | -2.44(-4.04,-0.83) | -1.91(-3.9,0.08) | -2.6(-4.54,-0.66) | -1.94(-3.37,-0.52) | -0.94(-2.26,0.39) | -0.22(-1.43,0.98) |

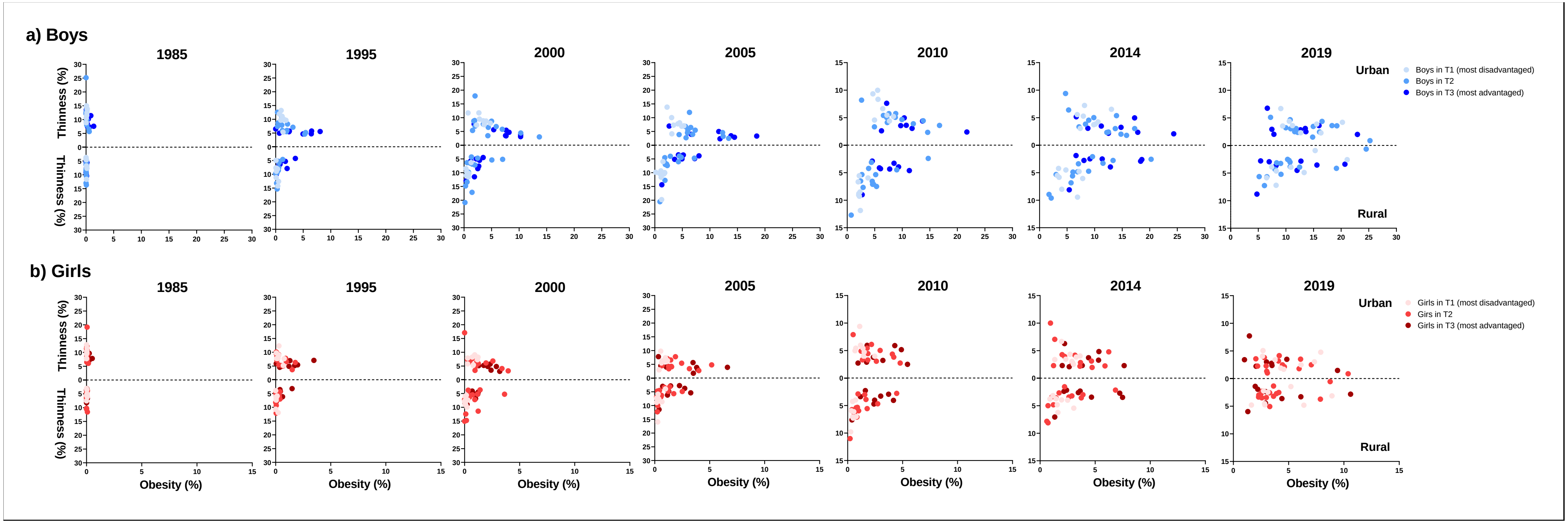


**Figure S1.** Secular trends in the prevalence of obesity and thinness at the provincial level, for both boys (a) and girls (b), stratified by urban-rural residence, 1985-2019.

*Note:* Each dot represents a province, with the horizontal axis depicting obesity prevalence and the vertical axis illustrating thinness prevalence. In terms of regional socioeconomic status, the color of these dots progresses from light to dark within each survey year, with blue representing boys and red representing girls. T1, the most disadvantaged socioeconomic status regions; T2, the moderate socioeconomic status regions; T3, the most advantaged socioeconomic status regions.

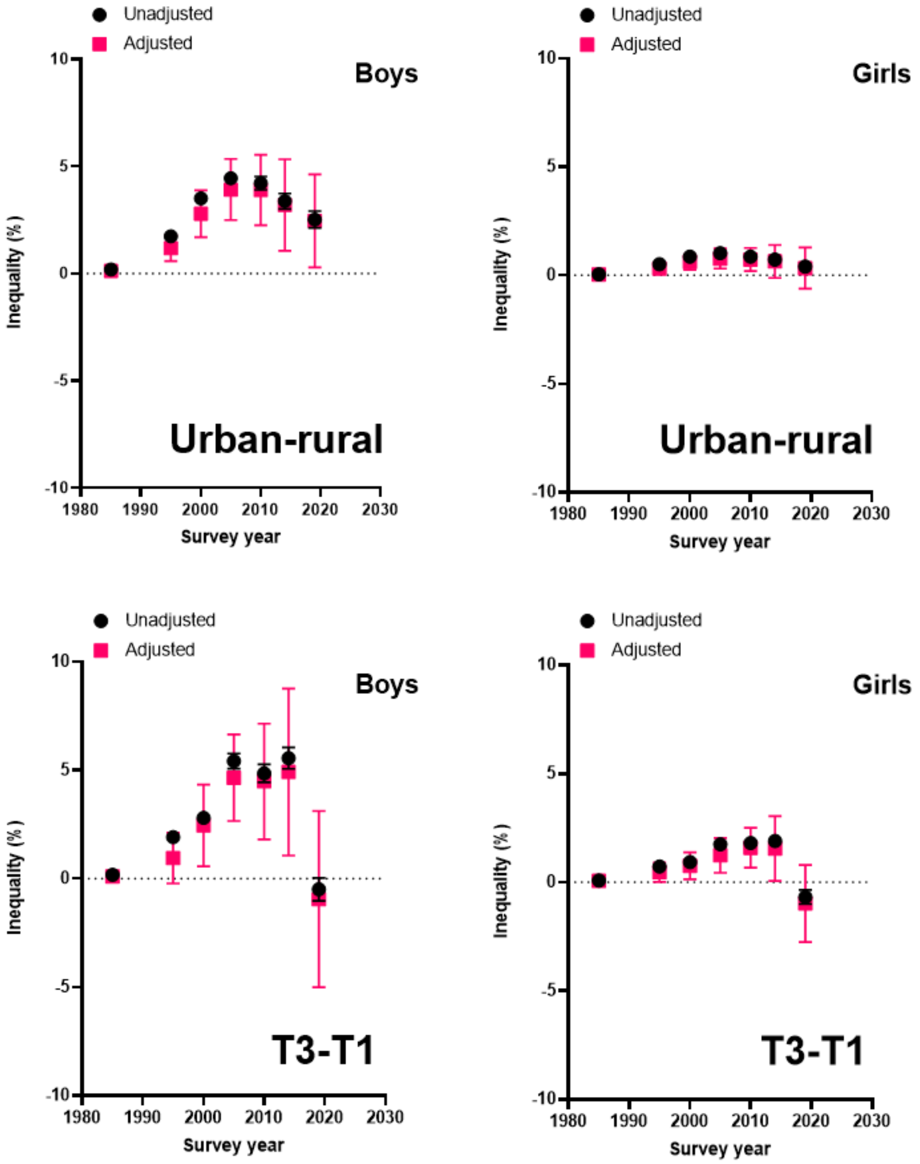


**Figure S2. Absolute urban-rural and regional socioeconomic inequalities (%) in childhood adolescent obesity, by sex**

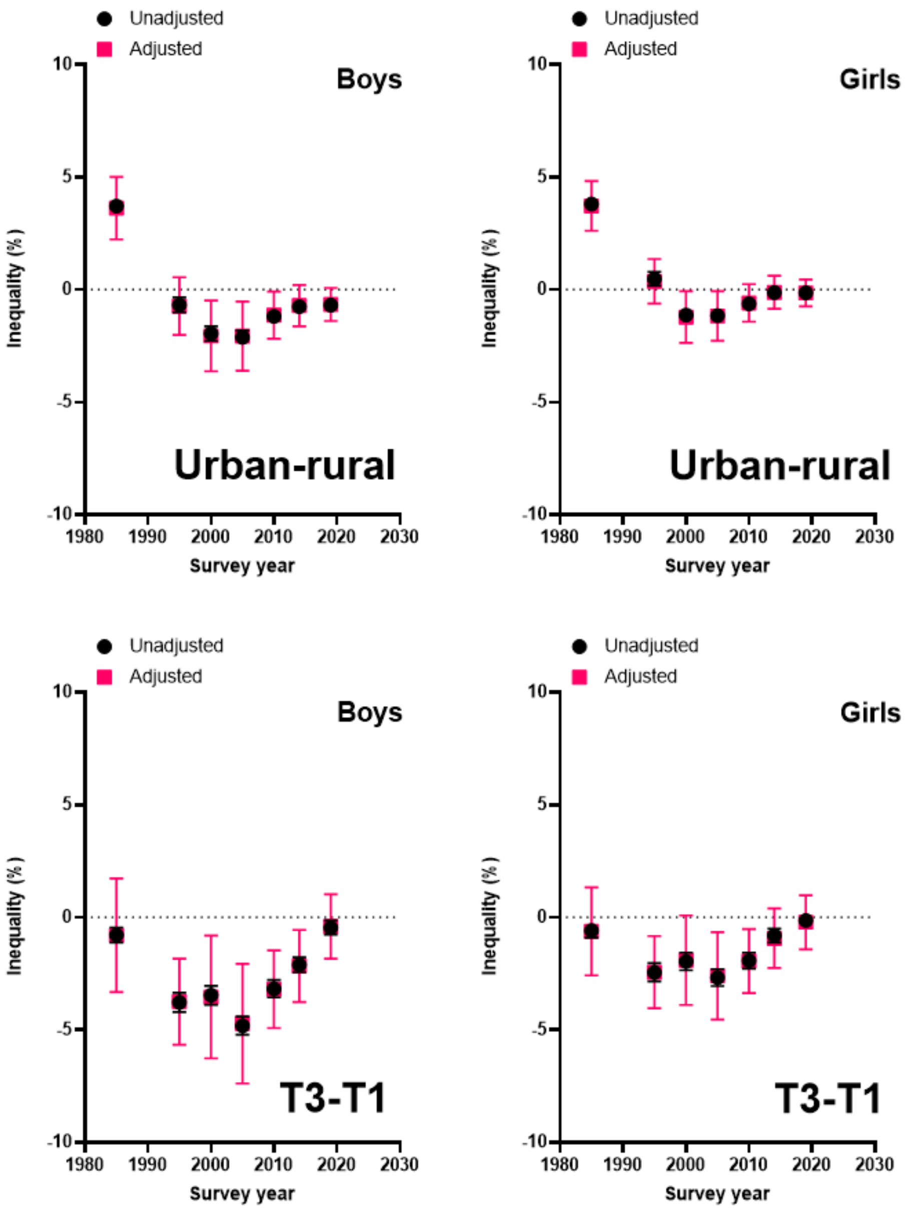


**Figure S3. Absolute urban-rural and regional socioeconomic inequalities (%) in childhood adolescent thinness, by sex**
